# Visualizing RNA Structures by SAXS-Driven MD Simulations

**DOI:** 10.3389/fbinf.2022.781949

**Published:** 2022-02-18

**Authors:** Weiwei He, Anja Henning-Knechtel, Serdal Kirmizialtin

**Affiliations:** ^1^ Chemistry Program, Science Division, New York University Abu Dhabi, Abu Dhabi, United Arab Emirates; ^2^ Department of Chemistry, New York University, New York, NY, United States

**Keywords:** SAXS (small-angle X-ray scattering), RNA, MD simulation, structural modelling, experimentally guided simulations, maximum entropy

## Abstract

The biological role of biomolecules is intimately linked to their structural dynamics. Experimental or computational techniques alone are often insufficient to determine accurate structural ensembles in atomic detail. We use all-atom molecular dynamics (MD) simulations and couple it to small-angle X-ray scattering (SAXS) experiments to resolve the structural dynamics of RNA molecules. To accomplish this task, we utilize a set of re-weighting and biasing techniques tailored for RNA molecules. To showcase our approach, we study two RNA molecules: a riboswitch that shows structural variations upon ligand binding, and a two-way junction RNA that displays structural heterogeneity and sensitivity to salt conditions. Integration of MD simulations and experiments allows the accurate construction of conformational ensembles of RNA molecules. We observe a dynamic change of the SAM-I riboswitch conformations depending on its binding partners. The binding of SAM and Mg^2+^ cations stabilizes the compact state. The absence of Mg^2+^ or SAM leads to the loss of tertiary contacts, resulting in a dramatic expansion of the riboswitch conformations. The sensitivity of RNA structures to the ionic strength demonstrates itself in the helix junction helix (HJH). The HJH shows non-monotonic compaction as the ionic strength increases. The physics-based picture derived from the experimentally guided MD simulations allows biophysical characterization of RNA molecules. All in all, SAXS-guided MD simulations offer great prospects for studying RNA structural dynamics.

## 1 Introduction

Living cells are composed of a high concentration of biomolecules. These molecules establish a complex network of interactions to maintain integrity and functionality (Sharp, 2009; Uversky et al., 2005; Yu et al., 2016). In addition to structure–function relationships (Pan and Sosnick, 2006; Lee et al., 2007), biomolecules perform its role by interacting with binding partners such as cofactors, ligands, and cations. Detailed knowledge about the structural dynamics coupled to the binding partners is important to elucidate their function and also develop therapeutics (Burnett and Rossi, 2012; Cully, 2018; Batool et al., 2019) and functional materials for nanotechnology (Jasinski et al., 2017; Seeman and Sleiman, 2017; Shi et al., 2017; Li Z. et al., 2020; Reuther et al., 2021).

Significant work has been invested in recent decades to determine the three-dimensional structure of biomolecules. The most notable methods have been X-ray crystallography, nuclear magnetic resonance (NMR) spectroscopy, and cryo–electron microscopy (cryo-EM) (Zheng et al., 2015; Nogales, 2016; Helliwell, 2017; Alderson and Kay, 2021). X-ray crystallography offers high atomic resolution of a dehydrated state of a biomolecule. NMR spectroscopy and cryo-EM, on the other hand, elucidate the solution structure of biomolecules. However, the biomolecules that can be investigated using these techniques are usually limited either by the experimental conditions or molecular size and flexibility. Another technique to study the structural dynamics of biomolecules is small and wide-angle X-ray scattering (SWAXS) (Petoukhov and Svergun, 2013; Kikhney and Svergun, 2015). The ease of sample preparation and the flexibility of studying the biomolecule understudy in a wide range of solvent conditions make SWAXS an important tool in investigating conformational ensembles of biomolecules in physiological conditions.

As far as RNA is concerned, it is difficult to obtain pure crystals for X-ray diffraction measurements. In addition, the crystallization conditions may impact the structure and cation hydration levels (DiGabriele et al., 1989; Ramakrishnan and Sundaralingam, 1993; Dickerson et al., 1994). For solution techniques, the high heterogeneity of the RNA conformations poses a challenge to structural interpretation. Typical properties of RNA molecules, such as small proton density, shorter transverse relaxation times, and low variance in its monomeric units, often bar the chemical shift dispersion during NMR (Varani et al., 1996; Allain and Varani, 1997; Tzakos et al., 2006). Likewise, despite recent efforts in RNA-only cryo-EM, it remains a challenge to resolve high-resolution structures of RNA molecules with accuracy on par with X-ray crystallography (Kappel et al., 2020; Zhang et al., 2021). The main challenge in SWAXS, on the other hand, is the lack of sharp spatial resolution (Hub, 2018).

Among computational approaches, molecular dynamics (MD) simulations serve as ideal tools to describe the structural dynamics of RNA molecules as they consider the RNA flexibility and coupling of RNA conformations with cation distributions in a physical way. However, obtaining an experimentally consistent description of RNA from simulations is still uncommon in the field due to the inaccuracies in the empirical potentials (Salsbury and Lemkul, 2021), as well as the challenges in sampling the rugged energy landscape of RNA conformations. Recent force field refinement efforts (Tan et al., 2018; Kührová et al., 2019; Mlýnský et al., 2020) hold promise for the accurate description of conformational ensembles of RNA molecules; yet, more work needs to be done to test the robustness of these refinements.

As it is not easy to obtain atomically detailed structural ensembles of RNA molecules neither in experiments nor in computer simulations that are necessary in understanding their biological function, a promising strategy to visualize RNA molecules has been carried out by integrating experiment and simulation (Jaynes, 1957; Kirmizialtin et al., 2012; Pitera and Chodera, 2012; Kirmizialtin et al., 2015; Bottaro and Lindorff–Larsen, 2018; Plumridge et al., 2018; Vendruscolo, 2018; Zhao and Shukla, 2018; Hermann and Hub, 2019; Li B. et al., 2020; Bottaro et al., 2020; Kirmizialtin et al., 2020; Orioli et al., 2020; Reißer et al., 2020; Shi et al., 2020; He et al., 2021; Bernetti et al., 2021). Here, data from various sources, such as NMR chemical shifts and SWAXS or fluorescence resonance energy transfer (FRET), can be introduced as constraints during simulations (Xu et al., 2008; Reinartz et al., 2018). A higher-resolution description of the biomolecules aimed to be obtained by this integration. In the restraining approach, the conformations are driven to satisfy experimental observations by applying additional biasing force (Chen and Hub, 2015; Hermann and Hub, 2019; Weiel et al., 2019; Larsen et al., 2020). This approach allows bypassing some of the sampling and force field issues (He et al., 2021). The experimentally consistent data, in turn, facilitate interpretation of the low-resolution signal. In re-weighting schemes, the already generated pool of conformations from simulations is re-weighted such that the average experimental signal is obtained. In this approach, the artifacts that may arise due to the restraining forces are avoided (Bottaro et al., 2018; Plumridge et al., 2018).

Here, we demonstrated the capability of small-angle X-ray scattering (SAXS)–driven MD simulations to study the RNA’s structure and dynamics. To showcase our method, we studied two RNA molecules: a riboswitch that is modulated by a ligand and metal ions, and a two-way junction RNA motif that shows sensitivity to ionic strength.

The S-adenosylmethionine (SAM)–sensitive riboswitch SAM-I regulates sulfur metabolism *via* the biosynthesis of sulfur-containing amino acids (Grundy and Henkin, 1998; Epshtein et al., 2003; Winkler et al., 2003). The binding of a SAM molecule to the RNA results in the formation of a terminator element, which turns off gene expression (McDaniel et al., 2003). This binding occurs in the presence of Mg^2+^ ions where the divalent cations stabilize the apo-state that facilitates the interaction with SAM. This state differs from the fully structured aptamer and, thus, is not eligible to switch off-gene expression. Although the crystal structure of SAM-bound aptamer is known (Montange and Batey, 2006), the atomically detailed structure of its apo-state(s) in the absence of SAM is not clearly understood. In addition, the role of Mg^2+^ ions on the structure and dynamics of the riboswitch is yet to be investigated. SAXS profiles at each functional state of the riboswitch are reported (Stoddard et al., 2010); however, the atomic level description of the structures demands more studies. Through the integration of SAXS experiments with all atom MD simulations, we will provide further insights into the molecular mechanism behind SAM-I riboswitch structural dynamics.

As a second system, we investigate a two-way junction, the so-called helix-junction-helix (HJH) RNA. There are many studies on their structural dynamics (Mustoe et al., 2012; Sutton and Pollack, 2015; Denny et al., 2018; Chen et al., 2019); however, the atomic details that are necessary to understand how these RNA molecules modulate their structural ensembles in different solvent conditions remain unclear. The structural elucidation of this important RNA motif has the potential to increase our knowledge about how RNA folds. We demonstrated in this work that the response of a two-way junction to its surroundings can be revealed using SAXS-driven MD.

## 2 Theory and Method

X-ray scattering measures the electron density contrast between the solute (in our case, RNA) and the solvent. All atom simulations provide detailed information for accurate computation of scattering (Park et al., 2009). Theoretically, the scattering from the instantaneous atom positions of solute system (*A*) that includes RNA, ions, and water in simulations can be computed from the electron density of the atoms in real space **R**, *A*(**R**) as follows:

$$A\left(q\right)=\int dR\;e^{-iq\cdot R}A\left(R\right),$$ (1)

where *A*(***q***) is the Fourier transform of the electron density of the coordinates and ***q*** is the scattering wave vector. The magnitude of *q* is determined by the scattering angle 2*θ* according to *q* = (4*π*/*λ*) *sin* *θ*, with the wavelength of the x-ray beam, *λ*.

The scattering intensity is then computed by

$$I_{A}\left(q\right)=\left\langle |A\left(q\right){|}^{2}\right\rangle .$$ (2)

Here, $\left\langle ‥\right\rangle$ represents the average over conformational ensemble, translational and rotational degrees of freedom.

In addition to the solute, the SAXS data include the scattering contribution of the solvent, which can be similarly computed as in Eqs 1–2, leading to $\left\langle B\left(q\right)\right\rangle$ . The simulated SAXS curves are then obtained by subtracting the buffer intensity (solvent system) from the scattering profile of the solute as

$$I_{com}\left(q,R\right)=\left\langle \langle |A\left(q\right){|}^{2}-\langle |B\left(q\right){|}^{2}\rangle \right\rangle .$$ (3)

### 2.1 Coupling SAXS Data With MD Simulations

The incorporation of the experimental SAXS data $\left[I_{\exp}\left(q\right)\right]$ to MD simulations (SAXS-driven MD) was achieved by adding an additional energy term to the Hamiltonian *E*_*hybrid*_ = *E*_*FF*_ + *E*_*SAXS*_, where *E*_*FF*_ is the energy from the MD force field, whereas *E*_*SAXS*_ represents the energetic penalty if the real-time computed amplitude $\left[I_{comp}\left(q\right)\right]$ deviates from the experimentally measured scattering curve $\left[I_{\exp}\left(q\right)\right]$ . The real-time SAXS curves are calculated on the fly from the simulation using the method described in by Chen and Hub (2015) and He et al. (2021). Typically, the energy penalty between the simulated conformations and the experimental data is evaluated by an uncertainty-weighted coupling term,

$$E_{SAXS}\left(R,I_{\exp},t\right)=\alpha \left(t\right)k_{c}\frac{k_{B}T}{n_{q}}\overset{n_{q}}{\underset{i=1}{\sum }}\frac{{\left\{\left[I_{com}\left(q_{i},R,t\right)\right]-\left[I_{\exp}\left(q_{i}\right)\right]\right\}}^{2}}{\sigma^{2}\left(q_{i}\right)},$$ (4)

where *σ* ^2^ (*q*_*i*_) is the experimental error given as $\sigma^{2}\left(q_{i}\right)=\sigma_{\exp}^{2}\left(q_{i}\right)+\sigma_{com}^{2}\left(q_{i}\right)+\sigma_{buffer}^{2}\left(q_{i}\right)$ that estimates the total error as the sum of experimental error, statistical error from computed curves, and systematic error from the uncertainty of the buffer density, respectively. $\left[I_{com}\left(q_{i},R,t\right)\right]$ is the real-time SAXS curve back-calculated from the simulation, which is the temporal-weighted average within a memory time of *t*, whereas *n*_*q*_ is the number of intensity points spanning the specific range of scattering vector *q*. The coefficient *k*_*c*_ is a constant that adjusts the weight of the SAXS potential *E*_*SAXS*_, as compared to the force field *E*_*FF*_ term. *k*_*B*_ and *T* denote the Boltzmann constant and temperature, respectively. Lastly, the parameter *α*(*t*) is a time-dependent function that allows a gradual introduction of the coupling potential at the start of the simulation.

### 2.2 Coupling Maximum Entropy Theory With SAXS-Driven MD Simulations

The coupling protocol in the previous section assumed that the ensemble could be dominated by a single, well-defined structure. This is often a useful approximation when the RNA molecule of interest has a known and well-defined structure such as double helix topologies of RNA and DNAs (He et al., 2021). However, some RNA molecules show structural heterogeneity. The resulted experimental signal, in our case, the SAXS profile, comes from a heterogeneous ensemble of distinct conformations. As a result, a conformational pool around one unique structure may be impossible to successfully satisfy the experimental restraints. The maximum entropy principle provides a unique and a minimally biased choice of restraint potential that can be used to bias the system for heterogeneous ensemble such that the experimental restraints are satisfied on average by introducing minimal unphysical artifacts (Pitera and Chodera, 2012). Introducing an experiment-derived biasing energy (*E*_*SAXS*_) into the simulation gives a biased ensemble *p*_*b*_(**R**) that represents the data that, according to the maximum entropy principle, are assumed to be as similar as possible to the original, unbiased ensemble *p*_*ub*_(**R**). The similarity could be mathematically evaluated by Kullback–Leibler divergence

$$D_{KL}\left(p_{b}|p_{ub}\right)=\int p_{b}\left(R\right)\ln\frac{p_{b}\left(R\right)}{p_{ub}\left(R\right)}dR.$$ (5)

Flexible structures are expected to adopt heterogeneous conformations; hence, the biased ensemble distribution *p*_*b*_(**R**) obtained by restraining a single simulation to the data (Eq. 4) deviates from the original ensemble distribution *p*_*ub*_(**R**), resulting in a high *D*_*KL*_, and that goes against the maximum entropy principle. To minimize the *D*_*KL*_, additional biasing terms of the back-calculated experimental data and the weight of each conformation are determined by Lagrange multipliers whose value is computed to enforce agreement between experiment and simulation.

To avoid the complications of determining the Lagrange multipliers, the replica-averaged modeling has been proposed (Best and Vendruscolo, 2004; Lindorff–Larsen et al., 2005). In this approach, a set of replicas of the system is simulated in parallel. The parallel-replica simulations with harmonic constraint to the ensemble-averaged data show to comply with the maximum entropy principle (parallel-replica SAXS-driven MD) (Pitera and Chodera, 2012; Roux and Weare, 2013). In this scheme, the simulated scattering curves are back-calculated by averaging the parallel replicas and giving more weight to the recent conformations (Chen and Hub, 2014)

$${\bar{I}}_{com}\left(q_{i},R_{1},\ldots ,R_{N},t\right)=N^{-1}\overset{N}{\underset{\beta =1}{\sum }}I_{com}\left(q_{i},R_{\beta },t\right),$$ (6)

where the replica index is denoted as *β*, so Eq. 4 of experiment-derived energy penalty is then modified accordingly

$$\begin{array}{cc} & E_{SAXS}\left(R_{1},\ldots ,R_{N},I_{\exp},t\right)\\ & =\alpha \left(t\right)k_{c}N^{\Omega }\frac{k_{B}T}{n_{q}}\overset{n_{q}}{\underset{i=1}{\sum }}\frac{{\left\{\left[{\bar{I}}_{com}\left(q_{i},R_{1},\ldots ,R_{N},t\right)\right]-\left[I_{\exp}\left(q_{i}\right)\right]\right\}}^{2}}{\sigma^{2}\left(q_{i}\right)} \end{array}$$ (7)

Following the works of Hummer and Köfinger (2015) and Hermann and Hub (2019), setting the exponent Ω in Eq. 7 to Ω = 1 ensures that the optimal ensemble is recovered as *N* → *∞*. Finally, it is to be noted that Eq. 4 and Eq. 7 share key parameters.

### 2.3 Molecular Modeling of SAM-I Riboswitch Aptamer

We study the SAM-I riboswitch aptamer at three solvent conditions. That is, (i) the SAM-bound and (ii) the SAM-free RNA molecule in the presence of Mg^2+^ ions (denoted henceforth as s1 and s2, respectively), and (iii) the Mg^2+^- and SAM-free state of the riboswitch (s3). We used for all these conditions the crystal structure of the SAM-bound complex (PDB entry: 2GIS) as the initial structure due to its good agreement with the reported SAXS data (Stoddard et al., 2010). The ligand was removed for the study of s2 and s3. Each of these starting coordinates was placed into a triclinic box with sufficient space to ensure structural changes of the RNA molecule during MD simulation toward the SAXS-based structural information. In detail, we selected a distance between the solute and the box of 1.2 and 4 nm for the conditions s1 and s2-3, respectively. The SAM-I riboswitch aptamer was solvated with water and ions to mimic the experimental condition of 7.6 mM MgCl_2_ and 150 mM NaCl. Additional 93 Na^+^ ions were added to satisfy charge neutralization. All these mono- and divalent ions were added to the simulation box by replacing water molecules. Energy minimization was employed to refine the structures using GROMACS 5.0.5. We used the *χ*OL3 force field (Zgarbová et al., 2011) to represent the RNA in combination with the TIP3P water model (Jorgensen et al., 1983). The force field was extended to include the topological information for SAM using the previous work (Hayes et al., 2012). The experimental SAXS curves show that the structure of the apo-state dramatically changes in the absence of *Mg* ^2+^ ions (s3). Hence, we used different starting structures. These starting structures were prepared by partially unfolding the RNA by separating the helices either through heating or pulling. For the heating simulations, the crystal structure was equilibrated in a 150 mM aqueous solution by running 5-ns-long simulation in an isothermal–isobaric (NPT) ensemble at 300 K, followed by a 20-ns-long canonical (NVT) ensemble at 350 K. To disrupt the specific tertiary contacts leading to the compact structures, we also applied force. After a 5-ns NPT simulation run at 300 K, the helices were pulled apart during a 20-ns NVT simulation with a pulling rate of 0.001 nm/ps and a force constant of 1,000 kJ/(mol × nm^2^). The pulling direction was defined as the x-component of the center of mass distance between P1 (nucleotides A6 and G89) and P3 (nucleotides U57 and C47). The two 20-ns-long trajectories were analyzed using the GROMOS algorithm with a cutoff of 0.2. The SAXS profile was calculated for each resulting cluster. The SAXS curve of seven structures was found to be close to the reported experimental SAXS curve for the Mg^2+^-free apo-state. Each of this structure was then energy minimized analogous to the s2 structure.

### 2.4 Molecular Modeling of RNA HJH

The starting structure of the RNA HJH construct, including two 12 base-paired RNA helices linked by a short chain of rU_5_, was built using Nucleic Acid Builder (NAB) (Case et al., 2005) by assuming an A-form geometry for the helix. The long backbone has a mixed sequence as CCC UAU ACU CCC UUU UUC CUC CUA AUC GC. The construct was placed in a cubic box by aligning the long axis parallel to the z-axis and energy minimized using GROMACS 5.0.5 with the same force parameters as the aforementioned ligand-free riboswitch system. The simulation box along the z axis was extended by about 20 Å at both ends of the RNA HJH to ensure that the molecule does not interact with its image. The resulting cubic box had a side length of 13.0 nm. The system was then solvated with TIP3P water and ions were added to match the experimental conditions. We studied the HJH system in the absence of K^+^ ions and to be consistent with experiment, in the presence of 50 mM, 100 mM, or 500 mM K^+^ concentration.

### 2.5 General MD Simulation Set up

All MD simulations of the riboswitch were carried out using the GROMACS 5.0.5 suite (Hess et al., 2008) with the imposed periodic boundary conditions in all directions. Possible bad contacts resulting from the random water and ion placement were removed by a 50,000-step long steep descent minimization. The minimized structures were then equilibrated for volume and solvent as follows: 5-ns-long simulation in isothermal–isobaric ensemble was employed with a constant temperature of 300 K and pressure of 1 bar ensured by using Berendsen thermostat (Berendsen et al., 1984) and Parrinello–Rahman barostat (Parrinello and Rahman, 1981), respectively. During this step, the heavy atoms of the RNA were restrained to their initial positions during the simulation, whereas ions and water were allowed to move freely. For this, harmonic restraints were applied to the RNA atoms with a force constant of 1,000 kJ/nm^2^. Particle mesh Ewald (PME) (Darden et al., 1993) summation was used to compute long-range electrostatic interaction with a grid spacing of 0.16 nm and an interpolation of order 4. The real space distance cutoff (for electrostatics and van der Waals energies) was set to 10-Å covalent bonds of the water and nucleic acid, which were constrained to their equilibrium geometries using SETTLE (Miyamoto and Kollman, 1992) and LINCS (Hess et al., 1997) algorithms, respectively. The equations of motion were solved using the Leapfrog scheme with a 1-fs time step. Subsequently, 20-ns constrained MD simulation was performed, using the dimensions and atom positions of the last snapshot of the NPT simulation. The settings for this run were kept the same as the previous section except that we turned the barostat off and the velocity scaling on.

For MD simulations of the RNA HJH, similar settings were used as for the riboswitch simulations. Here, we summarize the parameters that are different. For HJH, we used a PME set up with a grid spacing of 1.2 Å with an interpolation of order 4. The distance cutoff for non-bonded interactions and neighbor search was set to 11 Å. After equilibration, we performed 200 ns constrained MD at canonical ensemble during which heavy atoms of RNA were restrained, whereas water and ions were let free to move, and the output coordinate of this equilibration simulation was saved for the next step. To prepare the starting structures for the ensuing SAXS-driven simulations, we select conformations for each replica to reflect the structural diversity covering different regions of conformational space.

### 2.6 Computing SAXS From MD Trajectory

**FIGURE 1:**
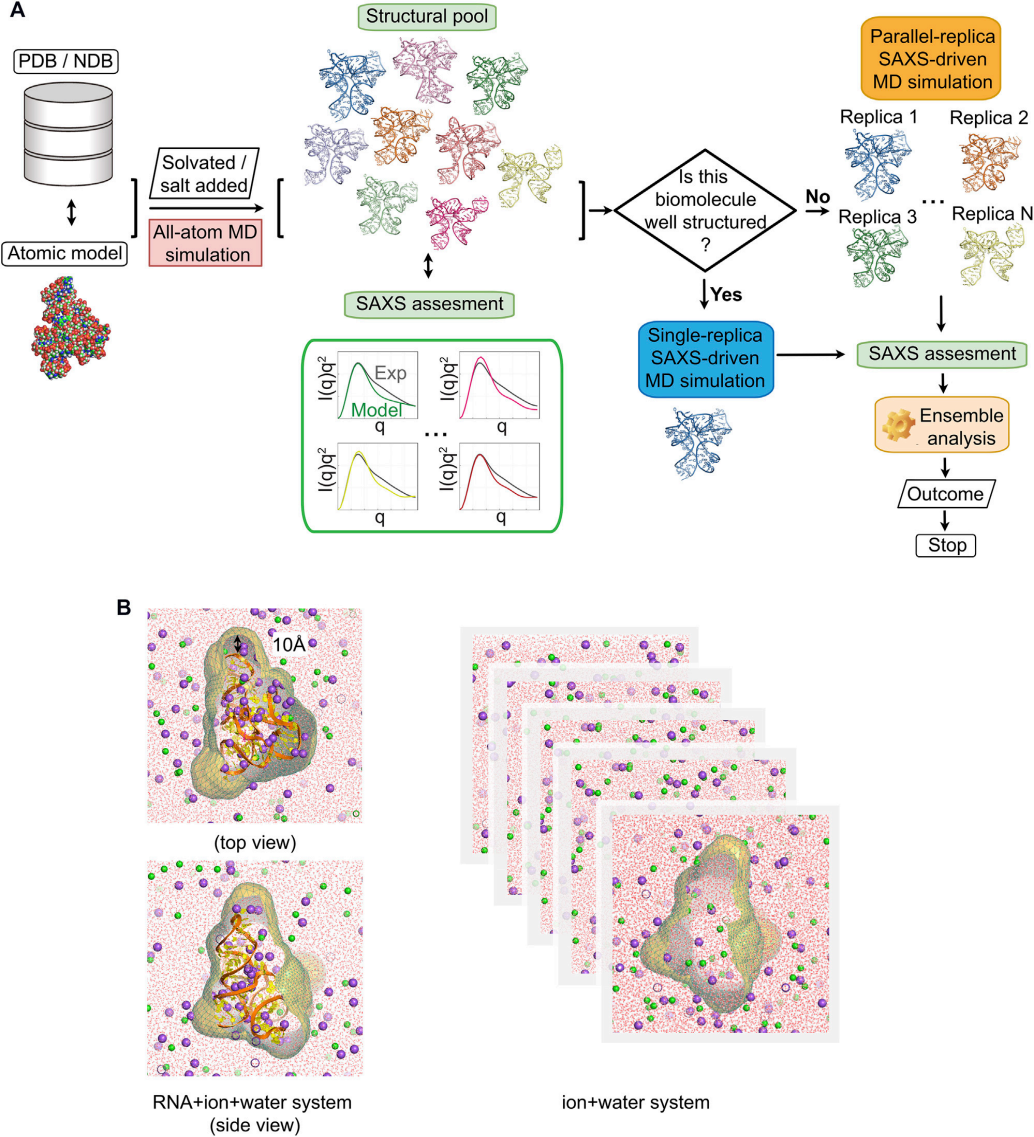
Flowchart of generating experimentally consistent RNA structures using small-angle x-ray scattering (SAXS) data. **(A)** The atomic model of target biomolecule is built or taken PDB/NDB. The step of the SAXS assessment after molecular modeling sorts the structure pool and picks out the best-fit conformations determined by the agreement (the threshold *χ* ^2^ ≤ 20). Different SAXS-driven strategies are used depending on RNA: a single-replica protocol for RNAs that are relatively well-structured, parallel-replica protocol for flexible RNA constructs. **(B)** Illustration of the RNA + ion + water and water + ion systems used to compute SAXS. The molecular envelope is constructed from a three-dimensional probability density isosurface 10 Å from the molecular surface to encompass the solvent/ion shell. The scattering intensity of the water + ion system was computed by applying the same envelope. We subtract the scattering from the RNA-ion system from solvent as described in Section 2.

The theoretical SAXS profiles were computed from the simulation according to the theory of excess electron density (Park et al., 2009; Chen and Hub, 2014) (see Eqs.1-3 in Section 2). By following the methods described previously (He et al., 2021), we built a spatial envelope to enclose the ion and water near the biomolecule with a distance cutoff of *d* = 10 Å from the RNA surface (see Figure 1B) to ensure a bulk-like solvent at the envelope isosurface. As shown in Figure 1B, on the basis of the solute system (RNA + ion + water), the molecular envelope was constructed and extended to bulk. In addition, to accurately estimate the electron density of the bulk solvent and approximate the excluded volumes, we ran 20-ns-long MD simulations of buffer environment at the NVT ensemble. The buffer simulation contains the ion pairs and water molecules in a periodic box with dimensions and concentration matching the solute system. Similar to the solute systems, an envelope that extends to 10 Å was applied to the solvent system for buffer subtractions.

### 2.7 The *χ* ^2^ Statistical Metric

The agreement between experimentally and computationally acquired SAXS profiles was quantitatively evaluated using the linear *χ* ^2^ metric

$$\chi^{2}=\frac{1}{n-1}\overset{n}{\underset{i=1}{\sum }}{\left\{\frac{\left[I_{\exp}\left(q_{i}\right)\right]-\left[I_{com}\left(q_{i}\right)\right]}{\sigma \left(q_{i}\right)}\right\}}^{2},$$ (8)

where *n* is the number of *q* points, and *I*_*com*_ (*q*_*i*_) and *I*_exp_ (*q*_*i*_) are the computed and experimental intensities at *q*_*i*_, respectively. In Eq. 8, *σ*(*q*_*i*_) is the experimental error.

### 2.8 SAXS-Driven MD Simulations Set up

In this work, we demonstrated two possible ways to couple the experimental information with MD simulations—a direct coupling approach based on harmonic restraints and a maximum entropy–based approach. We used the first approach to analyze the SAXS profile of the SAM-I riboswitch that is assumed to be dominated by a unique conformational state. In comparison, the structural interpretation of the flexible HJH was accomplished using the maximum entropy approach.

During SAXS-driven MD (Chen and Hub, 2015), we used stochastic dynamics (SD) integrator to sample conformations. We removed the center of mass motion to keep the RNA atoms at the box center, while maintaining other settings from the previous section of **GeneralMDsimulationsetup**. The scattering amplitudes were averaged by giving more weight to recent conformations using an exponential decaying memory function (Chen and Hub, 2014). The memory time *τ* was set to picoseconds and enabled the convergence of buffer subtraction over the time interval covering the fluctuations of solvent and the RNA backbone. A time interval *t* of 20 ns was used for the switch function *α*(*t*) to avoid strong SAXS-derived bias and to allow sufficient convergence of *I*_*com*_ (*q*, *t*) before applying the SAXS-derived forces.

The computed SAXS curves were recorded every 2.5 ps for analysis. The uncertainty of solvent density was set to 0.5*%*. We used 1,500 *q*-vectors for orientational average. The structures were refined using the experimental data upto *q* = 2.94 *nm* ^−1^, *q* = 3.15 *nm* ^−1^, and *q* = 3.15 *nm* ^−1^ for the models s1, s2, and s3, respectively. The number of restrained *q* points *n*_*q*_ was roughly determined according to the Shannon information theory (Shannon, 1949). We used *n*_*q*_ = 50 for our study on the SAM-I riboswitch structures. For the coupling potential, we chose *k*_*c*_ = 0.5. The convergence of simulations was evaluated frame by frame by *χ* ^2^ metric (Eq. 8). The conformations giving good agreement with experiments (*χ* ^2^ ≤ 2.0 in our case) are collected and used for further analysis.

Clustering of the data was done using the single linkage with a 0.1-nm cutoff, and the cluster centers were used to represent the corresponding ensembles. Root mean square fluctuation (rmsf) and the radius of gyration (Rg) of the RNA conformations, contact between the residues, and local *Mg* ^2+^ concentration were analyzed for conformations that show below *χ* ^2^ threshold value. In the case of structure s3b, this value was set to 15, for all other structures to 10.

In parallel-replica SAXS-driven MD (Hermann and Hub, 2019) of the RNA HJH, all settings are the same as single-replica simulations except for certain molecule-dependent parameters. As aforementioned, a four-replica protocol was applied in the study of HJH. We used a time interval *t* of 10 ns for the switch function *α*(*t*), which allows sufficient convergence of ${\bar{I}}_{com}\left(q,R_{1},\ldots ,R_{N},t\right)$ before introducing penalty. The structures were refined against the experimental data up to *q* = 2.9 *nm* ^−1^, and the coupling strength *k*_*c*_ was set to 1.0 in this case. We used 800 *q*-vectors for the orientational average of real-time SAXS calculation. We analyzed the convergence of simulation frame by frame using *χ* ^2^ metric and continued the simulation for at least another 100 ns upon reaching *χ* ^2^ threshold ( ≤ 1.5). The conformations giving good agreement with experiments (*χ* ^2^ ≤ 1.5) were selected and combined to build the conformational pool. The structures of the pool were clustered using the GROMOS (Daura et al., 1999) algorithm with a cutoff of 4.0 Å, and the cluster centers were used to represent each corresponding ensemble.

### 2.9 Computing Local *Mg* ^2+^ Concentration

To describe the local *Mg* ^2+^ concentration, we selected the phosphate group of each residue. The concentration around each phosphate group is estimated by the following expression (Nguyen and Thirumalai, 2020):

$$c\left(X_{\text{i}}\right)=g_{Xi}\left(r_{\max}\right)c_{bulk},$$ (9)

where *X*_*i*_ represents the phosphate group of residue *i*. *g*_*Xi*_ (*r*_max_) is the maximum peak height of the normalized radial distribution function (RDF) between the cation and phosphate atom(s) on the RNA, and *c*_*bulk*_ is the bulk solution concentration.

### 2.10 Computing FRET Efficiency of HJH From MD Trajectory

To cross-validate the sampled conformations, we computed the average FRET efficiency and compared with the experimental data. The theoretical FRET efficiency is estimated on the basis of the work of Schuler et al. (2005):

$$E_{FRET}=\int E\left(r\right)P\left(r\right)dr\;\;with\;\;E\left(r\right)={\left(1+{\left(r/R_{0}\right)}^{6}\right)}^{-1},$$ (10)

where R_0_ is set to be 60 Å after considering the linker length on the basis of previous works (Rothwell et al., 2003; Kalinin et al., 2010; Sindbert et al., 2011) and *P(r)* is the normalized distribution of distance (*r*) between the RNA surface where the FRET dyes attached in the experiments.

## 3 Results

Here, we explained our method of reconstructing the three-dimensional structures of RNA molecules from the SAXS data using atomic simulations. Figure 1 shows the flow chart of our approach. The pipeline starts with generating a structural pool that is ideally close to the experimentally sampled structural ensemble that we aim to resolve. Depending on the study, these initial models come from either a database [NDB(Berman et al., 1992) and PDB (Berman et al., 2000)], or other structural prediction tools (e.g., NAB and Rosetta FARFAR) (Lyskov et al., 2013; Watkins et al., 2020). We first computed the SAXS profile of each model to assess how close these models are to the experimental signal. We ranked the structures on the basis of the closeness of their computed SAXS profile to the experimental data. In this study, a *χ* ^2^ ≤ 30 is used to select potential candidates for the next step. In the second stage of the process, the selected initial models were prepared for MD simulations by adding explicit water and ions to mimic experimental conditions (see details in section Modeling and Simulations). The trajectory generated from MD simulations serves to assess the stability of the structure with an empirical potential. At the same time, MD simulations help to diversify the structural pool and to provide the solvation shell necessary to accurately compute the SAXS profiles (see details in Section 2.6). The SAXS computation method is schematically described in Figure 1B, which involves two systems: RNA in explicit ions and water and a buffer solution matching the same free cation concentration. After SAXS computation, the sample-and-select (SaS) strategy (He et al., 2021) serves to identify possible candidates that, in potential, are refined to match the experimental data. We select best-fit conformations from the pool by the criteria: *χ* ^2^ ≤ 20, and then, we applied SAXS-driven MD simulation to further refine structures. The method of choice for the SAXS-driven approach in this step depends on the inherent structural heterogeneity of the molecule under study and/or the uniqueness of the SAXS profile. If the RNA under study has a known unique fold and/or the RMSD of the RNA stays low ( ≤4 Å) during MD simulations, then we adopt single-replica SAXS-driven MD simulations. Here, the assumption is that the structure of the RNA is expected to adopt a single, well-defined structure in solution. Alternatively, if there are multiple folds that can explain the experimental SAXS profile and/or the RNA structure shows high flexibility, such as HJH, then we use multi-replica protocol to reduce the effect of the initial bias. The SAXS-driven simulations are monitored for their error-weighted sum of squared deviation. Conformations that give rise to *χ* ^2^ ≤ 2 are selected for the conformational pool for further analysis of the experimentally derived ensemble.

### 3.1 SAM-I Riboswitch

**FIGURE 2:**
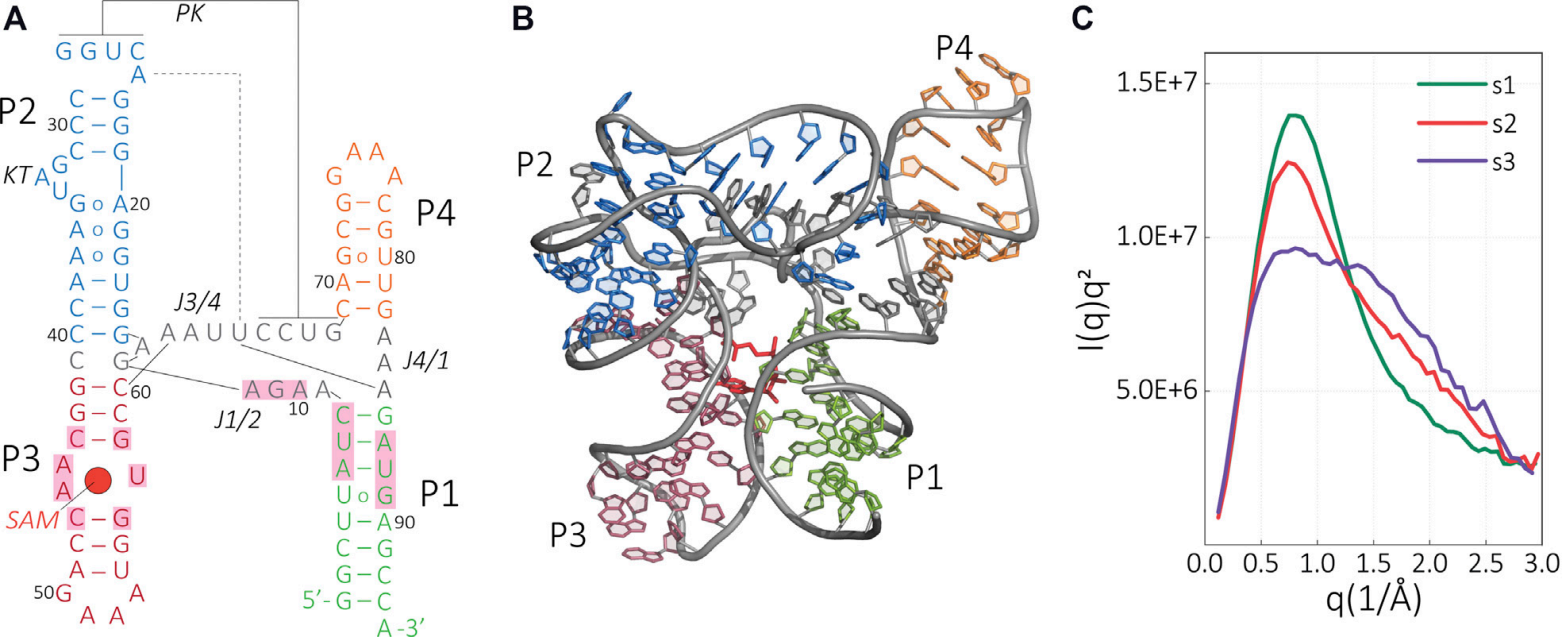
Structure and scattering profile of SAM-I riboswitch aptamer. **(A)** Secondary and **(B)** tertiary structure of the ligand-bound SAM-I aptamer. Key tertiary interactions are labeled as follows: P, paired; J, joining; KT, kink-turn; and PK, pseudoknot. The helices P1–P4 are colored in the tertiary structure according to the secondary structure representation. The ligand SAM is colored in red, and the SAM-binding residues are shaded in light red. **(C)** Kratky representation of the experimental scattering data that were used as a target structure for the SAXS-driven MD of the riboswitch under different configurations [s1: SAM(+),Mg^2+^(+); s2: SAM(−), Mg^2+^(+); s3: SAM(−), Mg^2+^(−)].

Using our methodology, we study the structural changes of metF-H2 SAM-I riboswitch from *Thermoanaerobacter tengcongensis*. In the presence of divalent ions and a ligand, this RNA folds into a four-way junction motif comprising a stem (P1; Figures 2A,B) and three stem-loop structures (P2–P4; Figures 2A,B). P1/P4 and P2/P3 form a co-axially stacked helix system where both helices are connected by a pseudoknot (PK) between P2 and P4, and joining regions J1/2 and J3/4. The linearity of P2 is interrupted by a kink-turn (KT), which facilitates the interactions in the PK region. As a result, further key tertiary contacts that connect P2 with J3/4 are formed. In addition, the crystal structure of the ligand bound complex shows a triple interaction between (A85–U64)•A24 and minor groove interactions between the P2 and A61-2 (Figure 2A). These network of interactions resulted in the three-dimensional structure shown in Figure 2B where the SAM ligand binds to a pocket that is situated between the helices P1 and P3 and the junction J1/2. The interaction of the adenine nucleobase of SAM with the asymmetric internal loop motif of the P3 helix is mainly characterized by hydrogen bonding, whereas the interaction with the minor groove of helix P1 is based on van der Waals surface complementarity. In addition, hydrogen bond formation can be found between the methionine moiety of SAM and J1/2.

Previous reported SAXS results by Stoddard et al. confirmed the similarity between the computed SAXS profile of the crystal structure of the ligand-bound SAM-I aptamer domain in the presence of Mg^2+^ with the solution SAXS profile (Stoddard et al., 2010). The profile of the solution structure was in good agreement with the theoretical SAXS curve derived from the crystal structure.

In addition to the ligand-bound structure, the SAXS study reports the aptamer structure in other important functional states, namely, the riboswitch without SAM in the presence of Mg^2+^ (apo-state, condition s2) and the apo-state without Mg^2+^ (condition s3). The reported Kratky plots of the scattering data show major differences depending on each of the conditions (Figure 2C). The changes in the SAXS profiles suggest structural differences in the riboswitch; however, an experiment-alone approach cannot resolve the atomic details.

**FIGURE 3:**
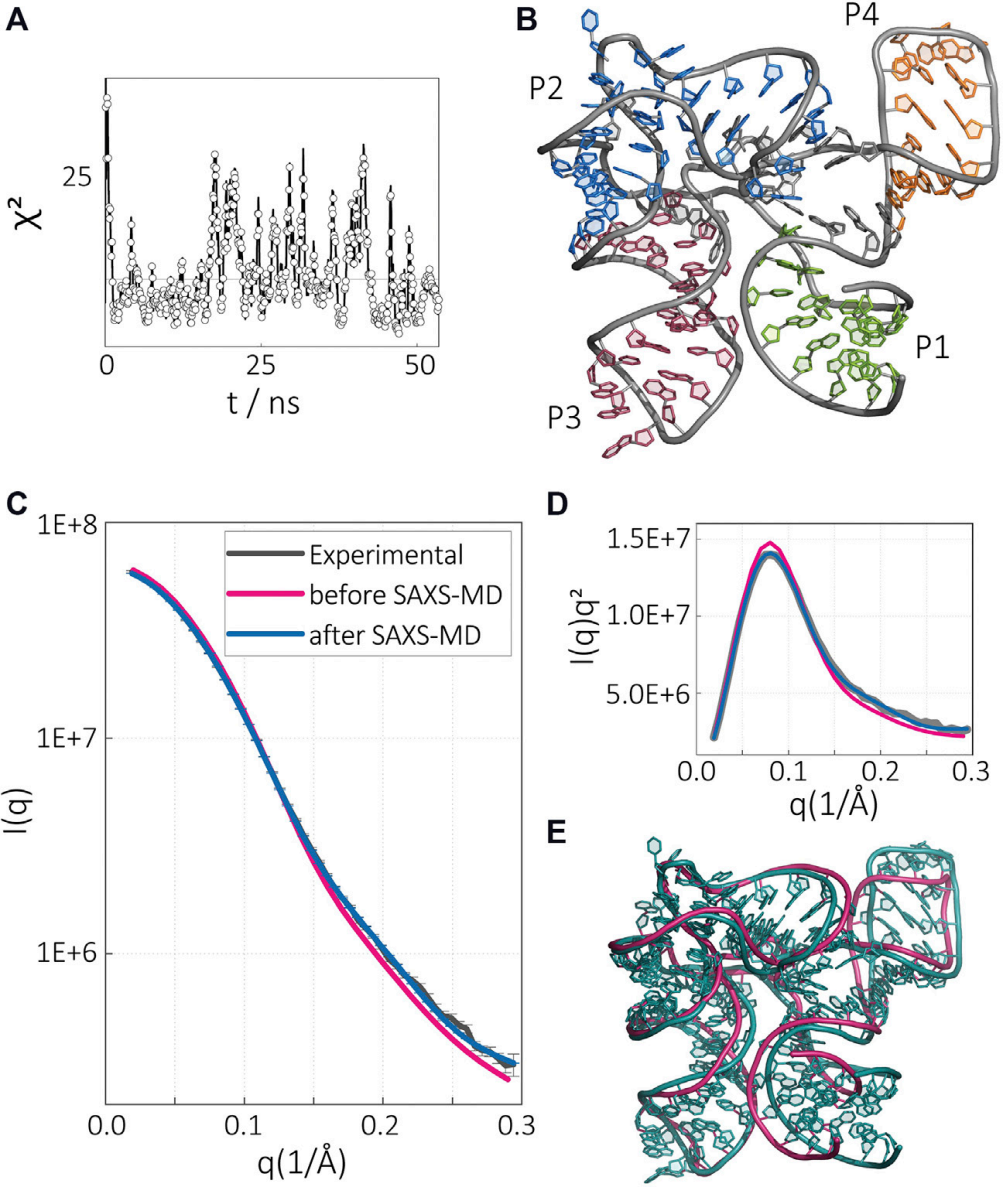
SAM-I riboswitch aptamer in the presence of SAM and Mg^2+^. **(A)** Time evolution of *χ* ^2^ variable during SAXS-driven MD simulation. **(B)** Converged structure after SAXS-driven MD simulation with experimental data for the Mg^2+^- and SAM-bound aptamer state. Intensity in the **(C)** normal and **(D)** Kratky representation. The experimental SAXS data (gray), computed SAXS data of the crystal structure (magenta), and computed SAXS data of the SAXS-driven MD final structure (blue). **(E)** Comparison of the SAXS-driven MD structure (blue) with the crystal structure (magenta).

To elucidate the implied structural differences reported in the SAXS profiles, we started with the SAXS profile of the SAM-bound riboswitch in the presence of Mg^2+^ (condition s1). Although the structure in this condition is already known, this exercise was aimed to establish our methodology and provide a benchmark against an independent data set. We have shown our results in Figure 3. Starting from the initial model adopted from the crystal structure, we computed the SAXS profile and monitor the error in SAXS as compared with the solution study. The *χ* ^2^ value for the crystal structure was about 30 at the beginning, but this value rapidly dropped to *χ* ^2^ ≈ 5 and started fluctuating around it, suggesting a convergence. We selected the lowest *χ* ^2^ value (1.88) structure as the representative of the RNA at this condition (Figure 3B). To compare the structure derived from our methodology, with the initial model and the experimental result, we compared the SAXS profiles (Figures 3C,D). The intensity profile averaged from our pool generated after the sampling converged shows excellent agreement with the experimental profile, whereas the profile computed from the crystal structure shows deviations at high *q* vectors suggesting subtle structural changes in the RNA at short pairwise distances in the solution condition. To resolve the differences, we overlay the representative solution structure to the crystal structure (Figure 3E). Our methodology suggests a slightly more expanded structure for the RNA in solution with minor structural changes localized in the P1/P4 stack (Figure 3E). This result is consistent with the expectations, as crystallization conditions are known to lead the compact states for biomolecules. This result also verifies our methodology and lays the foundation for further investigations of the apo-states that we will discuss below.

**FIGURE 4:**
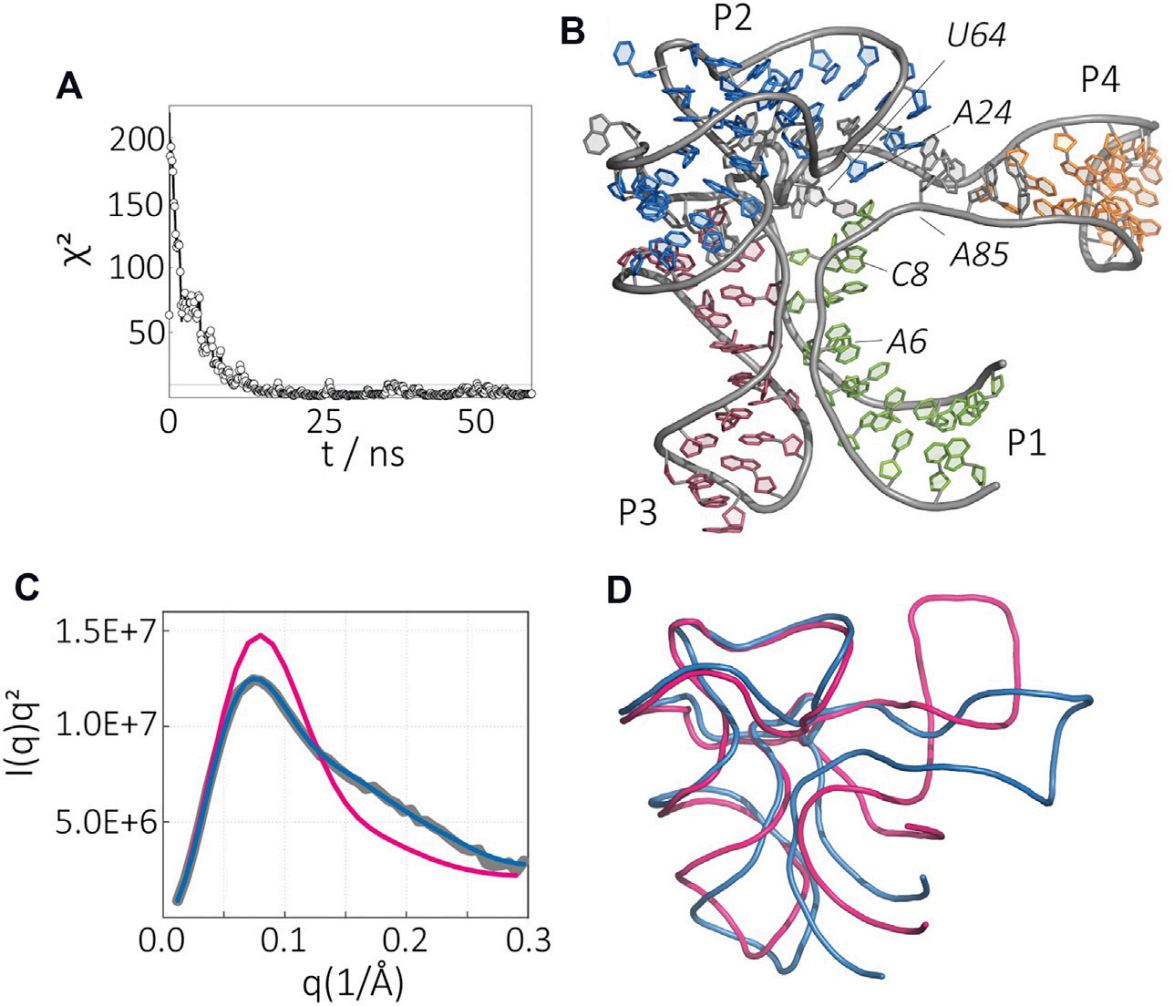
Apo-state of the SAM-I riboswitch aptamer in the presence of Mg^2+^. **(A)** Time evolution of *χ* ^2^ variable during simulation. **(B)** The final structure from SAXS-driven MD. **(C)** Kratky plot representation of the molecule, experimental SAXS data (gray), computed SAXS data of the crystal structure (magenta), and computed SAXS data of the SAXS-driven MD structure (blue). **(D)** Comparison of the SAXS-driven MD structure (blue) and the Mg^2+^-, and SAM-bound crystal structure (magenta).

Next, we show the structural ensemble of the apo-state of SAM-I riboswitch in the presence of Mg^2+^ (Figure 4). Time evolution of the error between experiment and simulation suggests a convergence after tens of nanoseconds. The lowest *χ* ^2^ reported here is 1.12. Figure 4B displays the best fit model, whereas Figure 4C compares the average SAXS profile of the ensemble (blue) with the experimental data (gray), contrasted with the SAXS profile of the initial model (red).

The atomic model of SAM-I riboswitch in the apo-state derived from SAXS-driven MD is depicted in Figure 4B. Marked differences are evident when overlaid with the SAM-bound state (Figure 4D.) In the apo-state, we observe an overall expansion of the riboswitch dimensions. Interestingly, most of the changes are localized in the P1/P4 stack region. The changes in the P1/P4 stack likely result in the loss of the tertiary interaction between P1 and P3 domains that can alter (A85–U64)•A24 triple interaction (Figure 4A). Although the interaction between P1 and P3 is lost, both helices remain close to each other, suggesting that the initial collapse of the RNA is due to electrostatic forces and the binding of magnesium ions.

**FIGURE 5:**
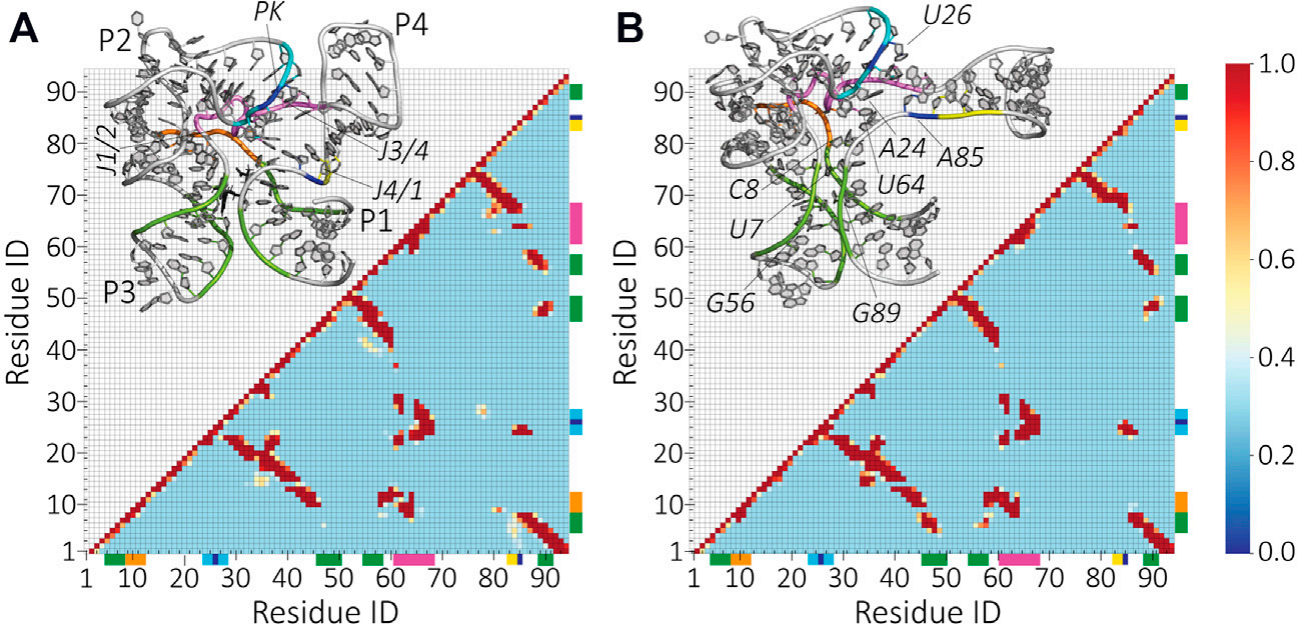
Contact formation frequency between the residues upon the removal of the ligand molecule SAM. Contact map of **(A)** the ligand-bound (s1) and **(B)** ligand-free (s2) SAM-I riboswitch. The map is based on a 6-Å cutoff for the minimum distance between the residues. Interhelical residues that get closer upon removing the ligand are highlighted with green and cyan/blue bars. The junctions J1/2, J3/4, and J4/1 are colored in orange, magenta, and blue-yellow, respectively. The same color scheme is applied in the inserted structural representation to illustrate the location of these contact changes.

To analyze the riboswitch structure in the ligand bound and unbound states in more detail, we compute the contact map from each ensemble. In Figure 5, we show the occupancy of contact pairs in s1 and s2 conditions. The important domains of the riboswitch and residues that show marked differences in contacts are highlighted in the insets. Interestingly, despite the notable differences in the SAXS profiles (Figure 2C), the close contact pairs show similar patterns. The absence of the ligand results in losing or gaining contacts. For example, the contacts between the PK-region of P2 and J4/1 are lost in the absence of SAM (residues highlighted in cyan-blue and blue-yellow). Instead, the residue A85 forms a new contact with residue U26 in the s2 condition (residues highlighted in blue). In addition, new contacts between P1 and P3 are formed (residues highlighted in green). These new contacts likely narrow down the SAM-binding pocket surrounded by residues A6-C8 and A46-C47, and G56-G58 and G89. The structural details revealed by our model suggest that SAM binds to an already formed binding pocket.

**FIGURE 6:**
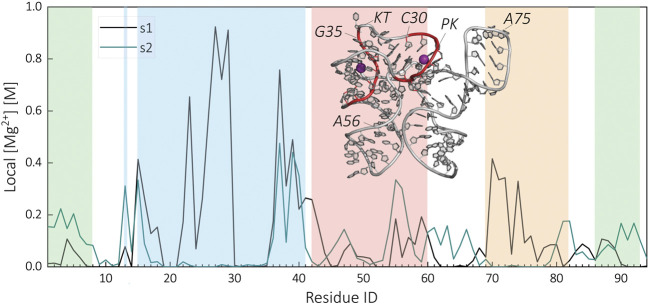
Comparison of the residue level local Mg^2+^ concentration at the ligand-bound (s1) and apo-state (s2) of the SAM-I riboswitch. The regions of the riboswitch are highlighted in color, P1 (green), P2 (blue), P3 (red), and P4 (orange). The inserted structural representation of s1 highlights the location of the two identified Mg^2+^ binding sites within the helix P2.

Our data show that the riboswitch in the presence of Mg^2+^ ions is able to stabilize most of its tertiary contacts in the apo-state. To gain insights into how Mg^2+^ ions are stabilizing the s2 state, we computed local Mg^2+^ ion concentration around the oxygen atoms of the phosphate group of each residue. We compared cation distributions in the s1 and s2 states. Figure 6 shows the variation in the local *Mg* ^2+^ concentration as a function of residue identities. On the basis of this analysis, we identified two major binding sites for s1. These binding sites are located around the P2 region. The first one is around the residues G22-C30, whereas the second binding site is around the G35-C40 downstream of the KT. The major change between s1 and s2 occurs around the PK region. The loss of contacts around G22-C30 leads to the diminishing of PK-Mg^2+^ binding site in the s2 state. In addition to the major binding sites, we observe binding sites specific to either s1 or s2. For example, in s1, Mg^2+^ ions show strong localization around A70-A75 due to the increased charge density in P4-P1 contact. This binding site is not present in s2 as the contact is lost. In contrast, in the s2 state, we observe an s2 specific binding site around A53-G56 due to the newly formed tertiary contacts between P1 and P3 domains.

A recent experimental study by Sarkar et al. identified two similar Mg^2+^ binding sites, around U26 and A36 (Sarkar et al., 2021). In addition, their study shows a decrease in Mg^2+^ association at U26 below a certain SAM concentration confirming the observed changes of the KT–Mg^2+^ interaction site in our study. However, we could not verify the Mg^2+^ binding site reported between J1/2 and J3/4 in the s1 state (Stoddard et al., 2010; Sarkar et al., 2021) in our simulations. This is most likely due to the limitations in our approach. As the prime focus of this study is the RNA structure, we did not run our simulations long enough to observe convergence in ion distributions. The chelated Mg binding sites require long simulation times. Further study is underway to investigate this issue.

Next, we investigated the structural ensemble of the riboswitch when there is no ligand and no Mg^2+^ ions are present (condition s3). As physiological conditions always have Mg^2+^ ions, this exercise helps us to further understand the functional role of Mg^2+^ ions in modulating the active structure of the riboswitch. The experimental data in the absence of Mg^2+^ ions (Figure 2C) indicate an open structure for RNA at this condition. For that purpose, a partially folded RNA that retains its secondary fold is assumed to be a good starting point to model this state. To obtain a suitable starting structure, we gently disrupt the tertiary contacts either by pulling the two helical stacks apart or heating the structure at 350 K. The resulting conformations were clustered, and the theoretical SAXS profile for each unique structure was calculated to identify candidate structures that may give rise to an agreement with the experimental data. The details of getting initial models are described in the Methods (Section 2.3).

**FIGURE 7:**
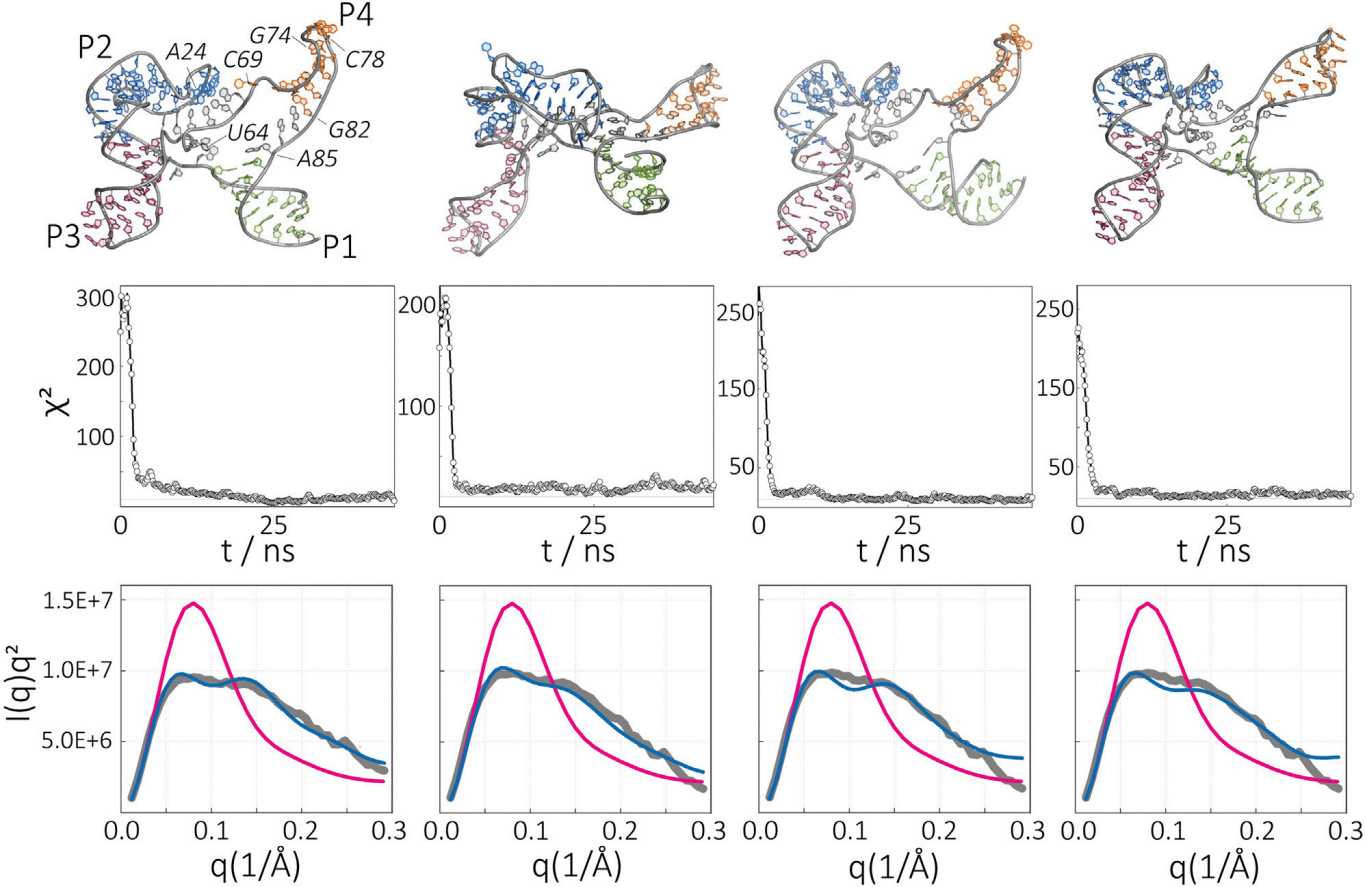
Apo-state of the SAM-I riboswitch aptamer in the absence of Mg^2+^. Ensembles of conformations obtained by SAXS-driven MD simulations. The final structures (top), time evolution of *χ* ^2^ (middle), and resultant Kratky plots (bottom) are given for four independent simulations. The intensity of the structure before (magenta) and after (blue) the SAXS-driven MD in comparison to the experimental data (gray).

From this structural pool, we selected about 10 structures and sampled conformations from each initial model independently using SAXS-driven MD simulations. Four of these structures reached a *χ* ^2^ value below a threshold (3a, *χ* ^2^ = 5.46; 3b, *χ* ^2^ = 12.35; 3c, *χ* ^2^ = 7.08; 3d, *χ* ^2^ = 7.39). The remaining simulations end up with high *χ* ^2^ and hence were discarded. The structures proposed by SAXS-driven MD and their Kratky plots are shown in Figure 7 in comparison to the experimental profile and the initial model. This result highlights the advantage of our approach, namely, the capability to obtain a structural ensemble that represents the experimental SAXS data instead of providing only one possible structure. This is especially important for RNAs that possess structural flexibility and conformational heterogeneity.

The absence of the ligand and *Mg* ^2+^ ions is reflected in the overall size of the RNA. The average radius of gyration, *R*_*g*_, measuring the overall size of a macroion shows a value of 2.14 ± 0.02 nm in the s1 state. This value increases to 2.39 ± 0.02 nm in the s2 state. Interestingly, the absence of binding partners leads to a major expansion of the structure, the *R*_*g*_ value becomes 2.75 ± 0.04 nm, in the s3 state. The resulting ensemble in the absence of binding partners is characterized by a larger gap between their P3 and P1 helices. The central part of the SAM-I riboswitch shows more open conformations. Similar to the s2 structure, here, the helix P4 is elongated. The similarities between s2 and s3 are likely due to a missing (A85–U64)•A24 triple interaction and the absence of short range interactions between helix P1 and P3 which happens in the absence of SAM. Despite the similarities between s2 and s3, there are additional changes unique to the s3 state; namely, an alternative base pairing of helix P4, the disruption of the base pairing between G82 and C69, and a 1-nt shorter loop structure due to an additional interaction between G74 and C78. As a result, P4 is less structured in the absence of *Mg* ^2+^ ions.

**FIGURE 8:**
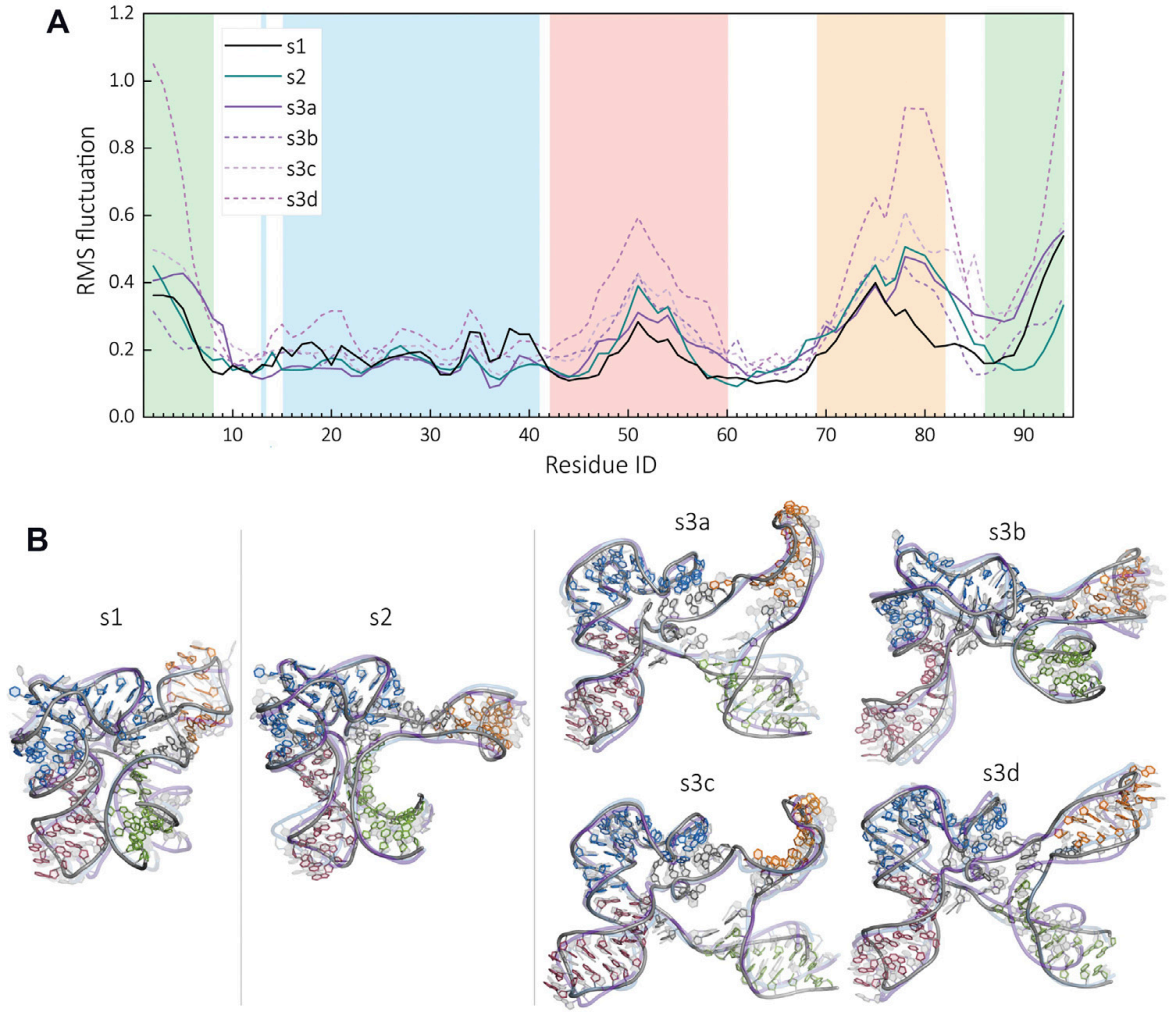
Comparison of the structural dynamics of SAM-I riboswitch at each functional states. **(A)** RMS fluctuations along the backbone of the RNA in the presence of SAM and Mg^2+^ (s1, black), in Mg^2+^ and without the ligand (s2, blue) and in the absence of divalent and SAM (s3, purple). The regions of the riboswitch, namely, P1, P2, P3, and P4, are highlighted in green, blue, red, and orange, respectively. **(B)** Structural dynamics is compared by showing the most populated clusters for each condition obtained by SAXS-MD.

The structural changes due to binding partners are also reflected in the dynamics of the riboswitch. To elucidate the dynamical differences in each condition, we monitored the residue level RMS fluctuations (Figure 8) from our conformational pool. The conformations representing different clusters aligned to display the mode of fluctuations (Figure 8B). The results indicate that the fluctuations decrease with higher compaction of the RNA molecules, due to the presence of *Mg* ^2+^ ions and SAM. The region with the highest fluctuations was under all conditions the 5′ and 3′ end of the RNA situated in helix P1, followed by the P4 domain, and the loop of P3. The flexibility of RNA shifts from the loop to the stem region of the P4 helix in the absence of the ligand. Interestingly, the PK region remains rigid in all conditions, with some minor differences within the P2 helix.

### 3.2 RNA Helix-Junction-Helix

**FIGURE 9:**
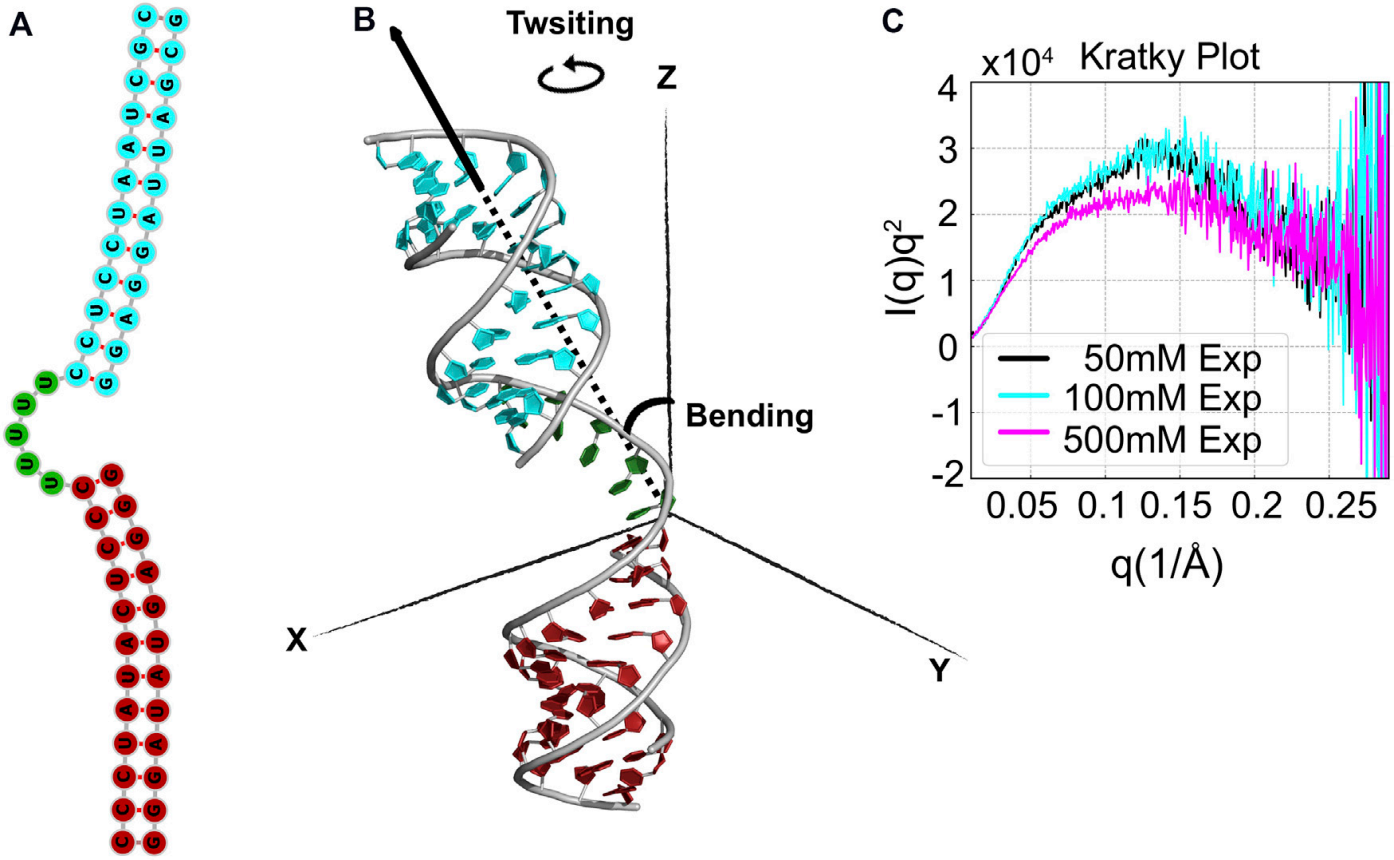
Structure and sequence of RNA HJH. **(A)** The HJH molecule consists of three components: 12-bp duplexes that are connected by a rU_5_ junction (green). **(B)** The positioning between the bottom duplex (cyan) and the upper duplex (firebrick) is used to define the two axial vectors measuring twisting and bending. **(C)** SAXS data from experiments in low (50 mM), middle (100 mM), and high (500 mM) [KCl] shown as Kratky plots.

Different from the riboswitch where the three-dimensional topology is relatively well restrained by tertiary contacts and ligands, the HJH motif (Figures 9A,B) possesses high structural heterogeneity. The flexible junction region allows its folding to be diverse, but at the same time, the base stacking of the junction region leads to distinct conformational sub-states. This high structural heterogeneity is modulated by solvent conditions, such as ion strengths and valence. Indeed, the conformations sampled in different salt conditions show detectable differences in the SAXS profiles for HJH RNAs (Chen et al., 2019; Chen and Pollack, 2019; Zettl et al., 2020; He et al., 2021).

Here, we investigate the conformational ensemble of HJH in varying ionic strengths of monovalent salt. In Figure 9C, we show the experimentally measured SAXS curves of RNA HJH represented by Kratky plots, *I*(*q*)*q* ^2^ vs. *q*, at 50 mM (low), 100 mM (medium), and 500 mM (high) [KCl]. Typically, the Kratky plots highlight the scattering features at high *q* up to 0.3Å^−1^. A more pronounced ridge in Kratky plots indicates the compactness of the molecule under study.

HJH’s sensitivity to salt concentrations is quite evident from the plots. Scattering profiles at low [KCl] and medium [KCl] show subtle deviations, especially at *q* ∈ (0.05 Å^−1^, 0.1 Å^−1^), which suggests conformational variations induced by increased [KCl]. Interestingly, such changes are amplified at high [KCl]. In particular, the reduction of the peak at mid-*q* regime [*q* ∈ (0.1 Å^−1^, 0.15 Å^−1^)] and the diminished peak at *q* ∈ (0.05 Å^−1^, 0.15 Å^−1^) indicates that the structure is even more extended at high-salt conditions. In other words, the raw SAXS data itself provide an interesting but counterintuitive observation—the cations are expected to soften electrostatic repulsion, which would lead to more compact states; however, the Kratky plot suggests more open conformations. The real-space projection of the SAXS profile could help to understand this observation.

**FIGURE 10:**
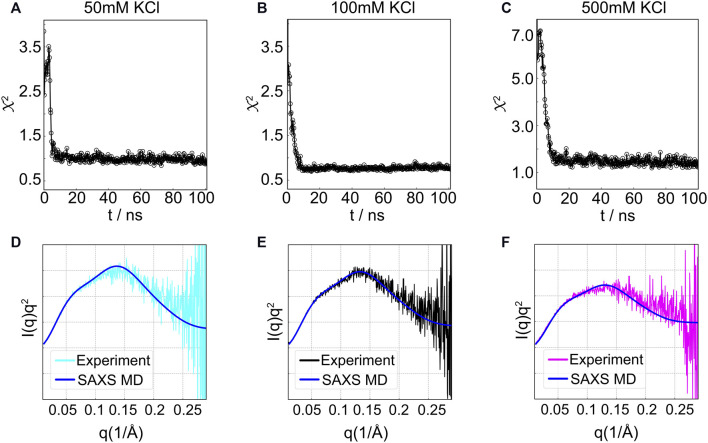
SAXS-driven MD simulation results for the HJH RNA at three concentrations. **(A–C)** Time evolution of *χ* ^2^ for **(A)** 50 mM, **(B)** 100 mM, or **(C)** 500 mM KCl. The asymptotic amplitude of *χ* ^2^ indicates the convergence. **(D–F)** Comparison of the measured SAXS data and the computed SAXS averaged over trajectory after the simulations converged.

By biasing simulations using the parallel-replica protocol (see details in Section 2.2), we obtained conformations that give rise to the experimentally consistent SAXS profiles. Here, we used four replicas as an optimum number to maximize the sampling and computational cost. Figure 10 summarizes the results for HJH simulations at different salt conditions. The structural search reached a plateau (*χ* ^2^ ≤ 1.5) within 20 ns (Figures 10A–C) with computed profiles that align well with the experimental data (Figures 10D–F) with a *χ* ^2^ value of 0.88, 0.85, and 1.35 for low, medium, and high [KCl], respectively. Interestingly, the *χ* ^2^ scores are lower than the previous study (Chen et al., 2019) using the ensemble optimization method (EOM) (Bernadó et al., 2007). The success is likely due to the fact that, in SAXS-driven MD, the structures are committed to agreeing with the experimental signal; whereas in the re-weighting approaches, the structures are selected from *a priori*–generated pool. The lack of overlap between the experimental and computational pool may lead to artifacts in re-weighting approaches.

**FIGURE 11:**
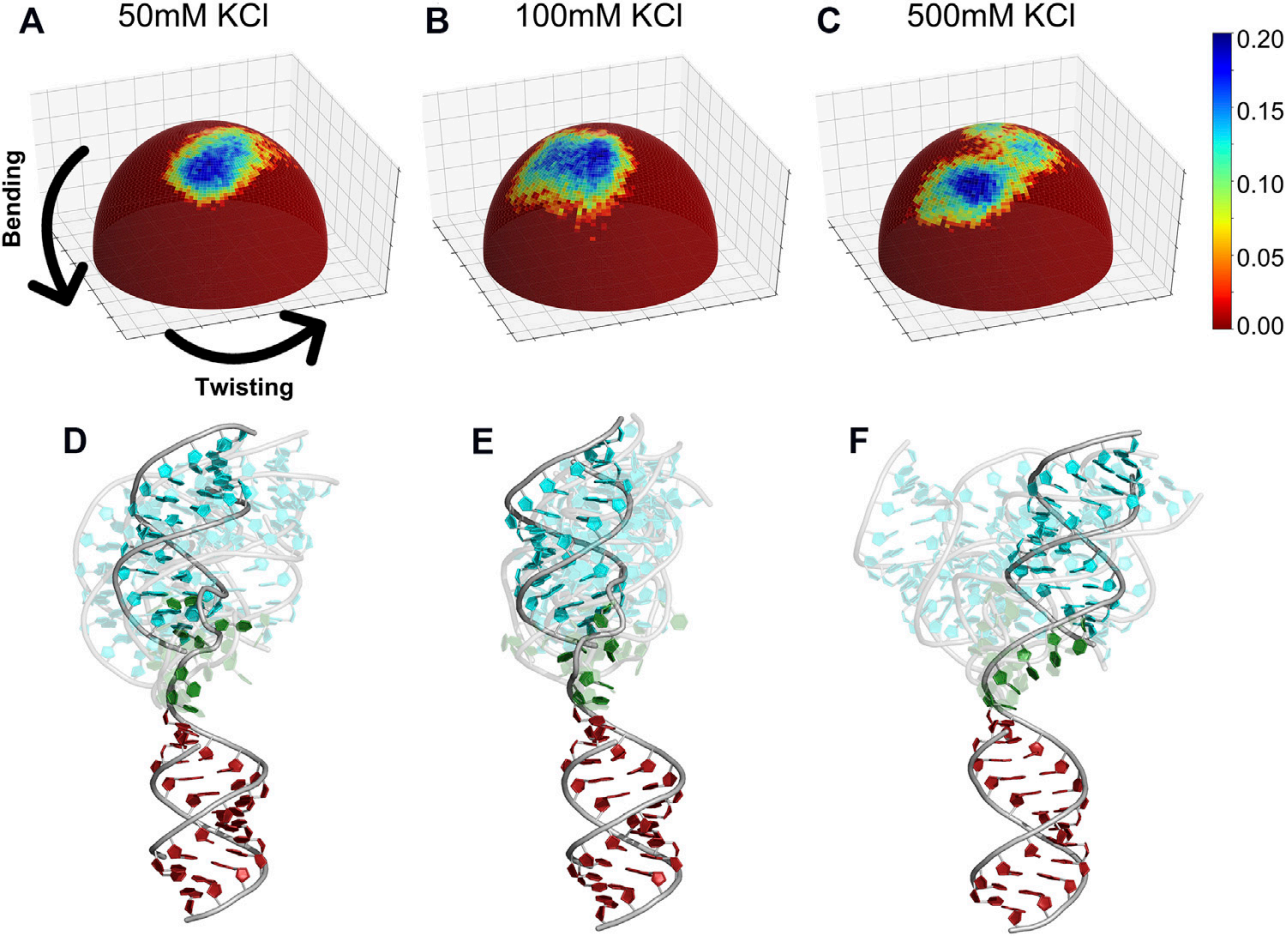
Spherical heat maps monitoring the occupancy of conformations visited by the HJH RNA. The conformations are monitored by two angles (bending and twisting) in different salt conditions **(A)** 50 mM, **(B)** 100 mM, and **(C)** 500 mM KCl. The representative structures **(D–F)** for each salt condition. Each frame represents a cluster center; the most populated cluster is colored in blue.

We then analyzed the trajectories of each salt condition that gives rise to good agreement with experiments. To visualize the structures, we align the bottom helix as shown in Figure 9B and project the HJH tip of the upper helix to a spherical coordinate system. The occupancy of conformations sampled is shown as a heat map (Figure 11). Interestingly, the conformations sampled by HJH show detectable differences depending on the ionic strength. In the low [KCl] (Figure 11A), we observed narrower distributions of helix orientations and less structural diversity reflected on the bending and twisting angles (see detailed definitions of orientation and bending in Figure 9B). At medium [KCl], the structural pool shifted to different bending angles retaining the twisting angle-similar to the low [KCl]. The conformations sampled at each salt condition are shown in (Figures 11D,E) for comparison. When it comes to high [KCl], however, the spherical heat maps reveal two distinct populations (Figure 11C). The HJH conformations now contain both extended (cyan) and bent states (blue) (Figure 11F). The bent states dominate the conformational pool at high-salt condition, yet the extended HJH states still contribute to the signal resulting in an average profile. Our visualization strategy resolves the conformational ensemble information hidden in the SAXS profiles. We conclude that the diminished peak at *q* ∈ (0.1 Å^−1^, 0.15 Å^−1^) of the SAXS curve is the result of this two co-existing populations.

**FIGURE 12:**
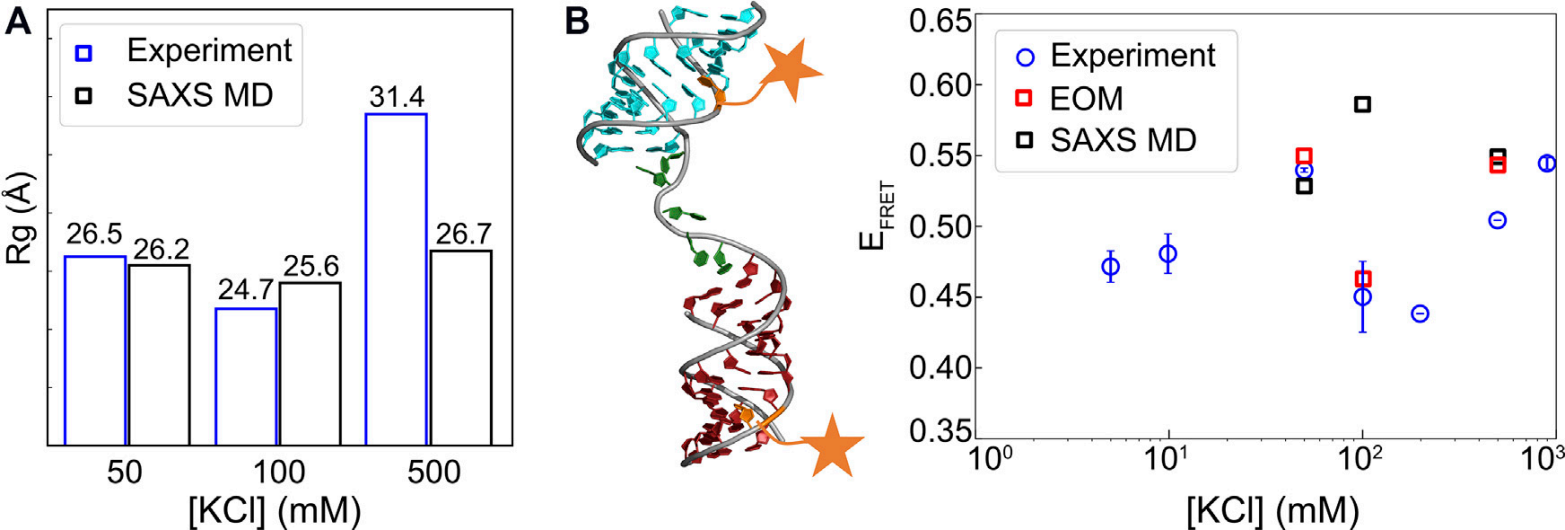
Comparison of radius of gyration (Rg) and efficiency of Förster resonance energy transfer (E_*FRET*_) values from experiment and modeling. **(A)** Rg from experiment and SAXS-driven MD and **(B)** FRET efficiencies from simulation and experiment. Fluorescent label sites are briefly illustrated by orange stars. R_0_ = 60 Å was applied for computing the theoretical E_*FRET*_ from MD simulations. The raw data and EOM analysis can be found in the works of Sutton and Pollack (2015) and Chen et al. (2019).

To further assess whether the conformations sampled by this approach lead to over-fitting, one has to check the predictions using independent data sets. For that purpose, we computed the *R*_*g*_ and FRET efficiency for cross-validation. The measurements of *R*_*g*_ and E_*FRET*_ are available for the HJH system under study which helps to test the accuracy of the conformational ensembles generated by our approach. Figures 12A,B show our comparisons. Remarkably, the model shows the trends of *R*_*g*_ consistent with experiments. At low [KCl], both descriptors give rise to good agreement, which indicates the success of our strategy in capturing the conformational ensemble of HJH at low salt. However, at medium salt, the E_*FRET*_ values that we computed deviate from the measurement although the *R*_*g*_ derived from the computational model is in good agreement with the measurements. At high-salt conditions, our prediction of *R*_*g*_ is lower than the measurement, yet E_*FRET*_ value computed from the simulation is comparable to the experimental measurement. The disagreement between FRET and SAXS at medium salt could be attributed to the added complexity by chromophores. Our simulation does not include the chromophores explicitly, so their interactions with each other and RNA are ignored, which might explain the differences between simulation and experiment. In addition, our calculations are based on Eq. 10, which assumes the orientation of dye pairs to be isotropic and uncorrelated. This assumption may not be valid especially when the dyes come close to each other, which is the case in medium salt. Another possibility for the discrepancy might stem from the differences in the sensitivity of FRET and SAXS. For HJH geometry, FRET shows a significant change in efficiency when the two helices rotate, maintaining the same distance. However, SAXS that reports only pairwise distances may not distinguish the rotation. Our method that relies on SAXS may miss the rotation leading to the discrepancy. Further study is needed to resolve this issue.

## 4 Discussion

Atomically detailed insights in RNA conformational ensembles are essential for understanding their function and developing strategies to utilize them. All-atom MD simulations that integrate SAXS can serve an important role in generating conformational pools consistent with experiments. In this study, we show that coupling of all-atom MD to SAXS data has the potential to extract the hidden information in the scattering profiles and provide an atomic visualization of the conformational dynamics that are otherwise impossible to interpret from experiment alone approaches.

Our analysis suggests that the presence of monovalent ions is sufficient for the riboswitch aptamer domain to fold into the helical stack structure and to maintain its secondary structure. The structure of the riboswitch in the absence of binding partners is open and highly flexible. The addition of Mg^2+^ ions and SAM rigidifies the domains by facilitating tertiary contacts, leading to a more organized RNA topology. Our findings on the SAM-I riboswitch strengthen the published study, where the SHAPE reactivity of the riboswitch aptamer revealed high mobility at the termini and the loop of P3 and P4 (Kirmizialtin et al., 2015). Similar to the study, we observe low mobility for P2. The picture gained from SAM-I riboswitch without the ligand also aligns well with the SHAPE studies; an elongation of P4 and a separation between the helices P1 and P3 are consistent with earlier works.

Concerning the flexible RNA HJH construct, we compared the simulated structures and corresponding microscopic properties at varying salt conditions. We observed extended states at low [KCl], which is due to the repulsive forces between the helices. The findings, including the cross-validation of *R*_*g*_ and E_*FRET*_, at low salt show a good agreement with the previously published studies (Chen et al., 2019). Although *R*_*g*_ or FRET analysis displays deviations between simulations and experiments at medium and high [KCl], our approach shows parallels with the previous works employed EOM methodology (Chen et al., 2019); yet, some minor differences between the two approaches may arise due to the differences in the sampled pool.

Visualization of RNA structures from experiments is not a trivial task. Despite the developments in recent years, the robustness of methods for combining MD and experimental data remains unsettled. The success of the methods depends on many factors. The resolution and distinctness of the experimental curves need to be verified for each system. The magnitude and type of errors in the experiment also play an important role. One of the main challenges is the low resolution of SAXS measurements. In SAXS measurements, the distance correlations can only reach sub-nanometer scales that do not provide high resolution to further refine the RNA structure. The application of wide angles has the potential to increase the resolution. Further technological advancements are needed to reduce the errors observed at high *q* regions to allow more accurate structural refinement.

In addition to experimental challenges, the outcome of experimentally driven computational simulation may depend on factors like the methodology implemented to integrate the simulation and experiment (Vendruscolo, 2018). It is yet not clear whether the maximum parsimony–based approaches such as EOM or the maximum entropy–based approaches, used here, better represent the actual RNA conformational ensemble measured by SAXS. Secondly, one has to check the quality of the back-calculation. Accurate computation of SAXS from simulations is still an ongoing research problem for RNA systems. In addition, the success of re-weighting schemes heavily relies on whether there is an overlap between the experimental ensemble and the simulated one. Methods to assess this overlap still remain to be established. For methods that apply biasing potentials, the quality of the chosen force field plays a significant role in the final conformational pool. Further, cross-validation of the results with independent experimental descriptors is crucial for the reliability of the visualization method described here. Finally, for all approaches aforementioned, a robust and rigorous conformational sampling and a good choice of starting structures are crucial. Further progress needs to be made in these directions to allow the accurate visualization of RNA molecules using SAXS as an experimental constraint.

## Data Availability

The original contributions presented in the study are included in the article/supplementary material; further inquiries can be directed to the corresponding author.

## References

[B1] Alderson T. R. Kay L. E. (2021). NMR Spectroscopy Captures the Essential Role of Dynamics in Regulating Biomolecular Function. Cell 184, 577–595. 10.1016/j.cell.2020.12.034 33545034

[B2] Allain F. H. Varani G. (1997). How Accurately and Precisely Can RNA Structure Be Determined by NMR. J. Mol. Biol. 267, 338–351. 10.1006/jmbi.1996.0855 9096230

[B3] Batool M. Ahmad B. Choi S. (2019). A Structure-Based Drug Discovery Paradigm. Int. J. Mol. Sci. 20, E2783. 10.3390/ijms20112783 31174387 PMC6601033

[B4] Berendsen H. J. C. Postma J. P. M. van Gunsteren W. F. DiNola A. Haak J. R. (1984). Molecular Dynamics with Coupling to an External bath. J. Chem. Phys. 81, 3684–3690. 10.1063/1.448118

[B5] Berman H. M. Olson W. K. Beveridge D. L. Westbrook J. Gelbin A. Demeny T. (1992). The Nucleic Acid Database. A Comprehensive Relational Database of Three-Dimensional Structures of Nucleic Acids. Biophys. J. 63, 751–759. 10.1016/S0006-3495(92)81649-1 1384741 PMC1262208

[B6] Berman H. M. Westbrook J. Feng Z. Gilliland G. Bhat T. N. Weissig H. (2000). The Protein Data Bank. Nucleic Acids Res. 28, 235–242. 10.1093/nar/28.1.235 10592235 PMC102472

[B7] Bernadó P. Mylonas E. Petoukhov M. V. Blackledge M. Svergun D. I. (2007). Structural Characterization of Flexible Proteins Using Small-Angle X-ray Scattering. J. Am. Chem. Soc. 129, 5656–5664. 10.1021/ja069124n 17411046

[B8] Bernetti M. Hall K. B. Bussi G. (2021). Reweighting of Molecular Simulations with Explicit-Solvent SAXS Restraints Elucidates Ion-dependent RNA Ensembles. Nucleic Acids Res. 49, e84. 10.1093/nar/gkab459 34107023 PMC8373061

[B9] Best R. B. Vendruscolo M. (2004). Determination of Protein Structures Consistent with NMR Order Parameters. J. Am. Chem. Soc. 126, 8090–8091. 10.1021/ja0396955 15225030

[B10] Bottaro S. Bussi G. Kennedy S. D. Turner D. H. Lindorff-Larsen K. (2018). Conformational Ensembles of RNA Oligonucleotides from Integrating NMR and Molecular Simulations. Sci. Adv. 4, eaar8521. 10.1126/sciadv.aar8521 29795785 PMC5959319

[B11] Bottaro S. Lindorff-Larsen K. (2018). Biophysical Experiments and Biomolecular Simulations: A Perfect Match. Science 361, 355–360. 10.1126/science.aat4010 30049874

[B12] Bottaro S. Nichols P. J. Vögeli B. Parrinello M. Lindorff-Larsen K. (2020). Integrating NMR and Simulations Reveals Motions in the UUCG Tetraloop. Nucleic Acids Res. 48, 5839–5848. 10.1093/nar/gkaa399 32427326 PMC7293013

[B13] Burnett J. C. Rossi J. J. (2012). RNA-based Therapeutics: Current Progress and Future Prospects. Chem. Biol. 19, 60–71. 10.1016/j.chembiol.2011.12.008 22284355 PMC3269031

[B14] Case D. A. Cheatham T. E. III Darden T. Gohlke H. Luo R. Merz K. M. Jr (2005). The Amber Biomolecular Simulation Programs. J. Comput. Chem. 26, 1668–1688. 10.1002/jcc.20290 16200636 PMC1989667

[B15] Chen P. C. Hub J. S. (2015). Interpretation of Solution X-Ray Scattering by Explicit-Solvent Molecular Dynamics. Biophys. J. 108, 2573–2584. 10.1016/j.bpj.2015.03.062 25992735 PMC4457003

[B16] Chen P. C. Hub J. S. (2014). Validating Solution Ensembles from Molecular Dynamics Simulation by Wide-Angle X-ray Scattering Data. Biophys. J. 107, 435–447. 10.1016/j.bpj.2014.06.006 25028885 PMC4104061

[B17] Chen Y. L. Lee T. Elber R. Pollack L. (2019). Conformations of an RNA helix-junction-helix Construct Revealed by SAXS Refinement of MD Simulations. Biophys. J. 116, 19–30. 10.1016/j.bpj.2018.11.020 30558889 PMC6341278

[B18] Chen Y. L. Pollack L. (2019). Salt Dependence of A-form RNA Duplexes: Structures and Implications. J. Phys. Chem. B 123, 9773–9785. 10.1021/acs.jpcb.9b07502 31638810 PMC7068736

[B19] Cully M. (2018). Antifungal Drugs: Small Molecules Targeting a Tertiary RNA Structure Fight Fungi. Nat. Rev. Drug Discov. 17, 864. 10.1038/nrd.2018.205 30482965

[B20] Darden T. York D. Pedersen L. (1993). Particle Mesh Ewald: An N Log (N) Method for Ewald Sums in Large Systems. J. Chem. Phys. 98, 10089–10092. 10.1063/1.464397

[B21] Daura X. Gademann K. Jaun B. Seebach D. Van Gunsteren W. F. Mark A. E. (1999). Peptide Folding: when Simulation Meets experiment. Angew. Chem. Int. Ed. 38, 236–240. 10.1002/(sici)1521-3773(19990115)38:1/2<236:aid-anie236>3.0.co;2-m

[B22] Denny S. K. Bisaria N. Yesselman J. D. Das R. Herschlag D. Greenleaf W. J. (2018). High-throughput Investigation of Diverse junction Elements in RNA Tertiary Folding. Cell 174, 377–e20. 10.1016/j.cell.2018.05.038 29961580 PMC6053692

[B23] Dickerson R. E. Goodsell D. S. Neidle S. (1994). “he Tyranny of the lattice”. Proc. Natl. Acad. Sci. USA. 91, 3579–3583. 10.1073/pnas.91.9.3579 8170950 PMC43623

[B24] DiGabriele A. D. Sanderson M. R. Steitz T. A. (1989). Crystal Lattice Packing Is Important in Determining the bend of a DNA Dodecamer Containing an Adenine Tract. Proc. Natl. Acad. Sci. USA. 86, 1816–1820. 10.1073/pnas.86.6.1816 2928304 PMC286795

[B25] Epshtein V. Mironov A. S. Nudler E. (2003). The Riboswitch-Mediated Control of Sulfur Metabolism in Bacteria. Proc. Natl. Acad. Sci. U S A. 100, 5052–5056. 10.1073/pnas.0531307100 12702767 PMC154296

[B26] Grundy F. J. Henkin T. M. (1998). The S Box Regulon: a New Global Transcription Termination Control System for Methionine and Cysteine Biosynthesis Genes in Gram-Positive Bacteria. Mol. Microbiol. 30, 737–749. 10.1046/j.1365-2958.1998.01105.x 10094622

[B27] Hayes R. L. Noel J. K. Mohanty U. Whitford P. C. Hennelly S. P. Onuchic J. N. (2012). Magnesium Fluctuations Modulate RNA Dynamics in the SAM-I Riboswitch. J. Am. Chem. Soc. 134, 12043–12053. 10.1021/ja301454u 22612276 PMC3675279

[B28] He W. Chen Y. L. Pollack L. Kirmizialtin S. (2021). The Structural Plasticity of Nucleic Acid Duplexes Revealed by WAXS and MD. Sci. Adv. 7, eabf6106. 10.1126/sciadv.abf6106 33893104 PMC8064643

[B29] Helliwell J. R. (2017). New Developments in Crystallography: Exploring its Technology, Methods and Scope in the Molecular Biosciences. Biosci. Rep. 37, BSR20170204. 10.1042/BSR20170204 28572170 PMC6434086

[B30] Hermann M. R. Hub J. S. (2019). SAXS-restrained Ensemble Simulations of Intrinsically Disordered Proteins with Commitment to the Principle of Maximum Entropy. J. Chem. Theor. Comput 15, 5103–5115. 10.1021/acs.jctc.9b00338 31402649

[B31] Hess B. Kutzner C. Van Der Spoel D. Lindahl E. (2008). GROMACS 4: Algorithms for Highly Efficient, Load-Balanced, and Scalable Molecular Simulation. J. Chem. Theor. Comput 4, 435–447. 10.1021/ct700301q 26620784

[B32] Hess B. Bekker H. Berendsen H. J. C. Fraaije J. G. E. M. (1997). LINCS: a Linear Constraint Solver for Molecular Simulations. J. Comput. Chem. 18, 1463–1472. 10.1002/(sici)1096-987x(199709)18:12<1463:aid-jcc4>3.0.co;2-h

[B33] Hub J. S. (2018). Interpreting Solution X-ray Scattering Data Using Molecular Simulations. Curr. Opin. Struct. Biol. 49, 18–26. 10.1016/j.sbi.2017.11.002 29172147

[B34] Hummer G. Köfinger J. (2015). Bayesian Ensemble Refinement by Replica Simulations and Reweighting. J. Chem. Phys. 143, 243150. 10.1063/1.4937786 26723635

[B35] Jasinski D. Haque F. Binzel D. W. Guo P. (2017). Advancement of the Emerging Field of RNA Nanotechnology. ACS Nano 11, 1142–1164. 10.1021/acsnano.6b05737 28045501 PMC5333189

[B36] Jaynes E. T. (1957). Information Theory and Statistical Mechanics. Phys. Rev. 106, 620–630. 10.1103/physrev.106.620

[B37] Jorgensen W. L. Chandrasekhar J. Madura J. D. Impey R. W. Klein M. L. (1983). Comparison of Simple Potential Functions for Simulating Liquid Water. J. Chem. Phys. 79, 926–935. 10.1063/1.445869

[B38] Kalinin S. Sisamakis E. Magennis S. W. Felekyan S. Seidel C. A. (2010). On the Origin of Broadening of Single-Molecule FRET Efficiency Distributions beyond Shot Noise Limits. J. Phys. Chem. B 114, 6197–6206. 10.1021/jp100025v 20397670

[B39] Kappel K. Zhang K. Su Z. Watkins A. M. Kladwang W. Li S. (2020). Accelerated Cryo-EM-Guided Determination of Three-Dimensional RNA-Only Structures. Nat. Methods 17, 699–707. 10.1038/s41592-020-0878-9 32616928 PMC7386730

[B40] Kikhney A. G. Svergun D. I. (2015). A Practical Guide to Small Angle X-ray Scattering (SAXS) of Flexible and Intrinsically Disordered Proteins. FEBS Lett. 589, 2570–2577. 10.1016/j.febslet.2015.08.027 26320411

[B41] Kirmizialtin S. Hennelly S. P. Schug A. Onuchic J. N. Sanbonmatsu K. Y. (2015). Integrating Molecular Dynamics Simulations with Chemical Probing Experiments Using SHAPE-FIT. Methods Enzymol. 553, 215–234. 10.1016/bs.mie.2014.10.061 25726467 PMC4777697

[B42] Kirmizialtin S. Pabit S. A. Meisburger S. P. Pollack L. Elber R. (2012). RNA and its Ionic Cloud: Solution Scattering Experiments and Atomically Detailed Simulations. Biophys. J. 102, 819–828. 10.1016/j.bpj.2012.01.013 22385853 PMC3283807

[B43] Kirmizialtin S. Pitici F. Cardenas A. E. Elber R. Thirumalai D. (2020). Dramatic Shape Changes Occur as Cytochrome C Folds. J. Phys. Chem. B 124, 8240–8248. 10.1021/acs.jpcb.0c05802 32840372 PMC7908931

[B44] Kührová P. Mlýnský V. Zgarbová M. Krepl M. Bussi G. Best R. B. (2019). Improving the Performance of the Amber RNA Force Field by Tuning the Hydrogen-Bonding Interactions. J. Chem. Theor. Comput 15, 3288–3305. 10.1021/acs.jctc.8b00955 PMC7491206 30896943

[B45] Larsen A. H. Wang Y. Bottaro S. Grudinin S. Arleth L. Lindorff-Larsen K. (2020). Combining Molecular Dynamics Simulations with Small-Angle X-ray and Neutron Scattering Data to Study Multi-Domain Proteins in Solution. Plos Comput. Biol. 16, e1007870. 10.1371/journal.pcbi.1007870 32339173 PMC7205321

[B46] Lee D. Redfern O. Orengo C. (2007). Predicting Protein Function from Sequence and Structure. Nat. Rev. Mol. Cel Biol 8, 995–1005. 10.1038/nrm2281 18037900

[B47] Li Z. Wang C. Li J. Zhang J. Fan C. Willner I. (2020). Functional DNA Structures and Their Biomedical Applications. CCS Chem. 2 (5), 707–728. 10.31635/ccschem.020.202000236

[B48] Li B. Cao Y. Westhof E. Miao Z. (2020). Advances in RNA 3D Structure Modeling Using Experimental Data. Front. Genet. 11, 574485. 10.3389/fgene.2020.574485 33193680 PMC7649352

[B49] Lindorff-Larsen K. Best R. B. DePristo M. A. Dobson C. M. Vendruscolo M. (2005). Simultaneous Determination of Protein Structure and Dynamics. Nature 433, 128–132. 10.1038/nature03199 15650731

[B50] Lyskov S. Chou F. C. Conchúir S. Ó. Der B. S. Drew K. Kuroda D. (2013). Serverification of Molecular Modeling Applications: the Rosetta Online Server that Includes Everyone (ROSIE). PloS one 8, e63906. 10.1371/journal.pone.0063906 23717507 PMC3661552

[B51] McDaniel B. A. Grundy F. J. Artsimovitch I. Henkin T. M. (2003). Transcription Termination Control of the S Box System: Direct Measurement of S-Adenosylmethionine by the Leader RNA. Proc. Natl. Acad. Sci. U S A. 100, 3083–3088. 10.1073/pnas.0630422100 12626738 PMC152250

[B52] Miyamoto S. Kollman P. A. (1992). Settle: An Analytical Version of the SHAKE and RATTLE Algorithm for Rigid Water Models. J. Comput. Chem. 13, 952–962. 10.1002/jcc.540130805

[B53] Mlýnský V. Kührová P. Kühr T. Otyepka M. Bussi G. Banáš P. (2020). Fine-tuning of the AMBER RNA Force Field with a New Term Adjusting Interactions of Terminal Nucleotides. J. Chem. Theor. Comput 16, 3936–3946. 10.1021/acs.jctc.0c00228 32384244

[B54] Montange R. K. Batey R. T. (2006). Structure of the S-Adenosylmethionine Riboswitch Regulatory mRNA Element. Nature 441, 1172–1175. 10.1038/nature04819 16810258

[B55] Mustoe A. M. Bailor M. H. Teixeira R. M. Brooks C. L. III Al-Hashimi H. M. (2012). New Insights into the Fundamental Role of Topological Constraints as a Determinant of Two-Way junction Conformation. Nucleic Acids Res. 40, 892–904. 10.1093/nar/gkr751 21937512 PMC3258142

[B56] Nguyen H. T. Thirumalai D. (2020). Charge Density of Cation Determines Inner versus Outer Shell Coordination to Phosphate in RNA. J. Phys. Chem. B 124, 4114–4122. 10.1021/acs.jpcb.0c02371 32342689

[B57] Nogales E. (2016). The Development of Cryo-EM into a Mainstream Structural Biology Technique. Nat. Methods 13, 24–27. 10.1038/nmeth.3694 27110629 PMC4913480

[B58] Orioli S. Larsen A. H. Bottaro S. Lindorff-Larsen K. (2020). “How to Learn from Inconsistencies: Integrating Molecular Simulations with Experimental Data. Prog. Mol. Biol. Transl Sci. Vol. 170, 123–176. 10.1016/bs.pmbts.2019.12.006 32145944

[B59] Pan T. Sosnick T. (2006). RNA Folding during Transcription. Annu. Rev. Biophys. Biomol. Struct. 35, 161–175. 10.1146/annurev.biophys.35.040405.102053 16689632

[B60] Park S. Bardhan J. P. Roux B. Makowski L. (2009). Simulated X-ray Scattering of Protein Solutions Using Explicit-Solvent Models. J. Chem. Phys. 130, 134114. 10.1063/1.3099611 19355724 PMC2852435

[B61] Parrinello M. Rahman A. (1981). Polymorphic Transitions in Single Crystals: A New Molecular Dynamics Method. J. Appl. Phys. 52, 7182–7190. 10.1063/1.328693

[B62] Petoukhov M. V. Svergun D. I. (2013). Applications of Small-Angle X-ray Scattering to Biomacromolecular Solutions. Int. J. Biochem. Cel Biol 45, 429–437. 10.1016/j.biocel.2012.10.017 23142499

[B63] Pitera J. W. Chodera J. D. (2012). On the Use of Experimental Observations to Bias Simulated Ensembles. J. Chem. Theor. Comput 8, 3445–3451. 10.1021/ct300112v 26592995

[B64] Plumridge A. Katz A. M. Calvey G. D. Elber R. Kirmizialtin S. Pollack L. (2018). Revealing the Distinct Folding Phases of an RNA Three-helix junction. Nucleic Acids Res. 46, 7354–7365. 10.1093/nar/gky363 29762712 PMC6101490

[B65] Ramakrishnan B. Sundaralingam M. (1993). Crystal Packing Effects on A-DNA Helix Parameters: A Comparative Study of the Isoforms of the Tetragonal & Hexagonal Family of Octamers with Differing Base Sequences. J. Biomol. Struct. Dyn. 11, 11–26. 10.1080/07391102.1993.10508706 8216939

[B66] Reinartz I. Sinner C. Nettels D. Stucki-Buchli B. Stockmar F. Panek P. T. (2018). Simulation of FRET Dyes Allows Quantitative Comparison against Experimental Data. J. Chem. Phys. 148, 123321–321. 10.1063/1.5010434 29604831

[B67] Reißer S. Zucchelli S. Gustincich S. Bussi G. (2020). Conformational Ensembles of an RNA Hairpin Using Molecular Dynamics and Sparse NMR Data. Nucleic Acids Res. 48, 1164–1174. 10.1093/nar/gkz1184 31889193 PMC7026608

[B68] Reuther C. Catalano R. Salhotra A. Vemula V. Korten T. Diez S. (2021). Comparison of Actin- and Microtubule-Based Motility Systems for Application in Functional Nanodevices. New J. Phys. 23, 075007. 10.1088/1367-2630/ac10ce

[B69] Rothwell P. J. Berger S. Kensch O. Felekyan S. Antonik M. Wöhrl B. M. (2003). Multiparameter Single-Molecule Fluorescence Spectroscopy Reveals Heterogeneity of HIV-1 Reverse Transcriptase:primer/template Complexes. Proc. Natl. Acad. Sci. U S A. 100, 1655–1660. 10.1073/pnas.0434003100 12578980 PMC149888

[B70] Roux B. Weare J. (2013). On the Statistical Equivalence of Restrained-Ensemble Simulations with the Maximum Entropy Method. J. Chem. Phys. 138, 084107. 10.1063/1.4792208 23464140 PMC3598863

[B71] Salsbury A. M. Lemkul J. A. (2021). Recent Developments in Empirical Atomistic Force fields for Nucleic Acids and Applications to Studies of Folding and Dynamics. Curr. Opin. Struct. Biol. 67, 9–17. 10.1016/j.sbi.2020.08.003 32950937 PMC7965779

[B72] Sarkar R. Jaiswar A. Hennelly S. P. Onuchic J. N. Sanbonmatsu K. Y. Roy S. (2021). Chelated Magnesium Logic Gate Regulates Riboswitch Pseudoknot Formation. J. Phys. Chem. B. 125, 6479–6490. 10.1021/acs.jpcb.1c02467 34106719 PMC8988897

[B73] Schuler B. Lipman E. A. Steinbach P. J. Kumke M. Eaton W. A. (2005). Polyproline and the "spectroscopic Ruler" Revisited with Single-Molecule Fluorescence. Proc. Natl. Acad. Sci. U S A. 102, 2754–2759. 10.1073/pnas.0408164102 15699337 PMC549440

[B74] Seeman N. C. Sleiman H. F. (2017). DNA Nanotechnology. Nat. Rev. Mater. 3, 1–23. 10.1038/natrevmats.2017.68

[B75] Shannon C. E. (1949). Communication in the Presence of Noise. Proc. IRE 37, 10–21. 10.1109/jrproc.1949.232969

[B76] Sharp P. A. (2009). The Centrality of RNA. Cell 136, 577–580. 10.1016/j.cell.2009.02.007 19239877

[B77] Shi H. Rangadurai A. Abou Assi H. Roy R. Case D. A. Herschlag D. (2020). Rapid and Accurate Determination of Atomistic RNA Dynamic Ensemble Models Using NMR and Structure Prediction. Nat. Commun. 11, 1–14. 10.1038/s41467-020-19371-y 33139729 PMC7608651

[B78] Shi W. Friedman A. K. Baker L. A. (2017). Nanopore Sensing. Anal. Chem. 89, 157–188. 10.1021/acs.analchem.6b04260 28105845 PMC5316487

[B79] Sindbert S. Kalinin S. Nguyen H. Kienzler A. Clima L. Bannwarth W. (2011). Accurate Distance Determination of Nucleic Acids via Förster Resonance Energy Transfer: Implications of Dye Linker Length and Rigidity. J. Am. Chem. Soc. 133, 2463–2480. 10.1021/ja105725e 21291253

[B80] Stoddard C. D. Montange R. K. Hennelly S. P. Rambo R. P. Sanbonmatsu K. Y. Batey R. T. (2010). Free State Conformational Sampling of the SAM-I Riboswitch Aptamer Domain. Structure 18, 787–797. 10.1016/j.str.2010.04.006 20637415 PMC2917978

[B81] Sutton J. L. Pollack L. (2015). Tuning RNA Flexibility with helix Length and junction Sequence. Biophys. J. 109, 2644–2653. 10.1016/j.bpj.2015.10.039 26682821 PMC4699881

[B82] Tan D. Piana S. Dirks R. M. Shaw D. E. (2018). RNA Force Field with Accuracy Comparable to State-Of-The-Art Protein Force fields. Proc. Natl. Acad. Sci. U S A. 115, E1346–E1355. 10.1073/pnas.1713027115 29378935 PMC5816156

[B83] Tzakos A. G. Grace C. R. Lukavsky P. J. Riek R. (2006). NMR Techniques for Very Large Proteins and RNAs in Solution. Annu. Rev. Biophys. Biomol. Struct. 35, 319–342. 10.1146/annurev.biophys.35.040405.102034 16689639

[B84] Uversky V. N. Oldfield C. J. Dunker A. K. (2005). Showing Your ID: Intrinsic Disorder as an ID for Recognition, Regulation and Cell Signaling. J. Mol. Recognit 18, 343–384. 10.1002/jmr.747 16094605

[B85] Varani G. Aboul-ela F. Allain F. H.-T. (1996). NMR Investigation of RNA Structure. Prog. Nucl. Magn. Reson. Spectrosc. 29, 51–127. 10.1016/0079-6565(96)01028-x

[B86] Vendruscolo M. (2018). Principles of Protein Structural Ensemble Determination. Biophysical J. 114, 388a–389a. 10.1016/j.bpj.2017.11.2149 28063280

[B87] Watkins A. M. Rangan R. Das R. (2020). FARFAR2: Improved De Novo Rosetta Prediction of Complex Global RNA Folds. Structure 28, 963–e6. 10.1016/j.str.2020.05.011 32531203 PMC7415647

[B88] Weiel M. Reinartz I. Schug A. (2019). Rapid Interpretation of Small-Angle X-ray Scattering Data. Plos Comput. Biol. 15, e1006900. 10.1371/journal.pcbi.1006900 30901335 PMC6447237

[B89] Winkler W. C. Nahvi A. Sudarsan N. Barrick J. E. Breaker R. R. (2003). An mRNA Structure that Controls Gene Expression by Binding S-Adenosylmethionine. Nat. Struct. Biol. 10, 701–707. 10.1038/nsb967 12910260

[B90] Xu X. Reinle W. Hannemann F. Konarev P. V. Svergun D. I. Bernhardt R. (2008). Dynamics in a Pure Encounter Complex of Two Proteins Studied by Solution Scattering and Paramagnetic NMR Spectroscopy. J. Am. Chem. Soc. 130, 6395–6403. 10.1021/ja7101357 18439013

[B91] Yu I. Mori T. Ando T. Harada R. Jung J. Sugita Y. (2016). Biomolecular Interactions Modulate Macromolecular Structure and Dynamics in Atomistic Model of a Bacterial Cytoplasm. eLife 5, e19274. 10.7554/eLife.19274 27801646 PMC5089862

[B92] Zettl T. Shi X. Bonilla S. Sedlak S. M. Lipfert J. Herschlag D. (2020). The Structural Ensemble of a Holliday junction Determined by X-ray Scattering Interference. Nucleic Acids Res. 48, 8090–8098. 10.1093/nar/gkaa509 32597986 PMC7641307

[B93] Zgarbová M. Otyepka M. Šponer J. Mládek A. Banáš P. Cheatham T. E. (2011). Refinement of the Cornell et al. Nucleic Acids Force Field Based on Reference Quantum Chemical Calculations of Glycosidic Torsion Profiles. J. Chem. Theor. Comput 7, 2886–2902. 10.1021/ct200162x PMC3171997 21921995

[B94] Zhang K. Zheludev I. N. Hagey R. J. Haslecker R. Hou J. Y. Kretsch R. (2021). Cryo-EM and Antisense Targeting of the 28-kDa Frameshift Stimulation Element from the SARS-CoV-2 RNA Genome. Nat. Struct. Mol. Biol. 28, 747–754. 10.1038/s41594-021-00653-y 34426697 PMC8848339

[B95] Zhao C. Shukla D. (2018). SAXS-guided Enhanced Unbiased Sampling for Structure Determination of Proteins and Complexes. Sci. Rep. 8, 17748. 10.1038/s41598-018-36090-z 30531946 PMC6288155

[B96] Zheng H. Handing K. B. Zimmerman M. D. Shabalin I. G. Almo S. C. Minor W. (2015). X-ray Crystallography over the Past Decade for Novel Drug Discovery - where Are We Heading Next. Expert Opin. Drug Discov. 10, 975–989. 10.1517/17460441.2015.1061991 26177814 PMC4655606

